# Meeting Abstracts for the Society for Simulation in Europe 2023

**DOI:** 10.1186/s41077-023-00270-3

**Published:** 2024-02-29

## O1. A 10-year bibliometric analysis of Latin America and the Caribbean healthcare simulation studies: focus on top-10 production trends

### Victor Velásquez-Rimachi^1^, Fernando Runzer-Colmenares^1^, Miguel Cabanillas-Lazo^2^, Solange Dubreuil-Wakeham^1^, Daniela Samaniego-Lara^1^, Percy Mayta-Tristan^1^, Alvaro Priale-Zevallos^1^

#### ^1^Universidad Científica del Sur; ^2^Sociedad Científica de San Fernando

*Adv Simul 2023*, **8(1)**:O1

***Introduction***

Healthcare simulation (HS) in Latin America and the Caribbean (LAC) has presented a growing development over the last 20 years. However, its facilitators remain focused on correctly applying the method without sufficient registration and budget to carry out research as an effective way of communicating knowledge (1, 2). Our aim was to describe the bibliometric indicators of LAC-HS studies between 2012-2021 to identify areas of opportunity.

***Methods***

We use Scival tool to collect the bibliometric data from the Scopus database. We carried out a systematic search with terms related to “Simulation training” to identify studies in HS of any design or language from 2012 to 2021 in LAC. We excluded articles not included in the theme “medicine”.

***Results & Discussion***

We got 847 documents with 8620 citations (10.2 citations/document). Surgery was the most popular subcategory (*n*=231; 27.3%). In LAC countries, South American authors from Brazil (*n*=152) and Chile (*n*=56) published the most. However, Cuban and Jamaican publications have the highest impact, with 41.5 and 38 citations/document, respectively. In the rest of the world, most co-authors are from the USA (*n*=430). However, Swiss and Italian authors have the highest impact, with 23.8 and 23.6 citations/document, respectively (Table 1). “Universidade de São Paulo” has the highest scientific production (*n*=45; 5.3%) and the highest number of citations (*n*=400). The high concentration of LAC-simulation documents was published in Q1 journals (*n*=319; 37.7%). Most of the documents developed topics in “debriefing & clinical competence” (*n*=166), and “surgical skills” (*n*=157), with the greatest growth of 77.2% and 189%, respectively.

Most documents have collaborations: only national (*n*=324; 38.3%), international (*n*=277; 32.8%) or only institutional (*n*=212; 25.1%). However, in terms of impact, international collaboration (12.4 citation/document) exceeds both national plus institutional collaborations (9.4 citation/document). USA is the country with the highest occurrence of co-authorships with countries with at least 5 published documents.

***Keywords***

Simulation Training; Bibliometrics; Latin America; Caribbean Region

References and/or acknowledgements may be included in the Reference area.

***References***

1. Hoogenboom B, Manske R. Invited commentary how to write a scientific article. The International Journal of Sports Physical Therapy. 2012; 7 (5): 512-517.
2. Corvetto M, Rubio R. Investigación en Simulación en Latinoamérica: una buena y una mala noticia. Simulación Clínica. 2019;1(1):3-4. doi:10.35366/RSC191A.

**Table 1 (Abstract O1):**
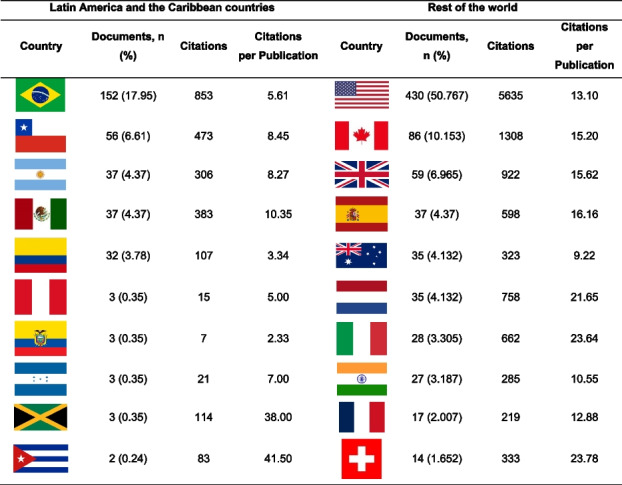
Top ten countries publishing on healthcare simulation in Latin America and the Caribbean

## O2. A systematic review of tools for clinical debriefing: attributes, evidence for use and validity evidence

Published article: https://qualitysafety.bmj.com/content/early/2023/03/27/bmjqs-2022-015464

*Adv Simul 2023*, **8(1)**:O2

## O3. Addressing our blindspots: medical students’ experiences of simulation-based education to support their recognition of implicit bias

### Sharan Mahtani^1^, Ian Thomas^1^, Helen Freeman^1^, Jess Gurney^2^, Jane Hislop^2^

#### ^1^NHS Highland; ^2^Edinburgh Medical School

*Adv Simul 2023*, **8(1)**:O3

***Introduction***

The GMC’s ‘Outcomes for Graduates’ states that newly qualified doctors should recognise and manage their own Implicit Biases (1). Implicit bias (IB) refers to attitudes unconsciously affecting our understanding, actions, and decisions (2).

Implicit Bias Recognition and Management (IBRM) strategies have included using online tests, lectures/workshops, and more recently Simulation-based education (SBE) (2). Literature suggests that SBE offers a unique approach to allow learners to recognise their own IB however more evidence is needed around SBE’s use in IB with medical students (3).

This study aimed to explore medical students’ experiences of an SBE session on recognition of IB and to see if the SBE session could facilitate transformative learning around IB. Insight from this could help inform how to best to utilise SBE in education around IB and how to integrate this into medical curricula.

***Methods***

This study involved interviews with volunteer fourth- and fifth-year medical students from the University of Aberdeen (UoA) who were recruited to undertake a SBE session and a post SBE interview.

A simulated ward round scenario was designed around a series of events which would expose learners to escalating IB triggers. The students’ experience of this and their recognition IB were explored through a debrief.

Following the SBE, participants were invited to attend a semi-structured interview on MS Teams where they were asked questions about their views, experiences and learning around IB.

The interviews were transcribed verbatim and transcripts were coded and analysed thematically. Ethical approval was granted by UoA Ethics Review Board (SERB/2021/12/2236).

***Results & Discussion***

Four themes have been identified from preliminary analysis (*n*=5) which are outlined in table 1. Findings suggest that the experience of the SBE validated participants’ own experiences of IB. Participants discussed the way that the SBE raised their awareness of IB and discussed the professional expectations of doctors to be unbiased. Finally, participants’ identified the challenges they felt when confronting and dealing with micro-aggressions.

This study set out to explore medical students’ experiences of a SBE around IB and whether this led to transformative learning around IB. Findings suggest that the SBE activity increased medical students’ recognition of IB in the workplace. This study is on-going and further interviews are planned. The findings are important to inform planning of curricula to ensure graduates are meeting the standards set out in the ‘Outcomes for Graduates’.

***Keywords***

Implicit Bias, Microaggressions, EDI, SBE

***References***

1. GMC. 2018. Outcomes for Graduates [Online]. General Medical Council. Available: Available at:
2. https://www.gmc-uk.org/media/documents/dc11326-outcomes-for-graduates-2018_pdf-75040796.pdf [Accessed 26th October 2021].
3. Sukhera, J. and Watling, C., 2018. A framework for integrating implicit bias recognition into health professions education. Academic Medicine, 93(1), pp.35-40.
4. Vora, S., Dahlen, B., Adler, M., Kessler, D.O., Jones, V.F., Kimble, S. and Calhoun, A. Recommendations and Guidelines for the Use of Simulation to Address Structural Racism and Implicit Bias. Simul Healthc. 2021 Aug 1;16(4):275-284.

**Table 1 (Abstract O3):** Themes and illustrating quotes

**Theme**	**Quotation**
Validating experience	Participant 1: “As a future BAME doctor myself, it was validating for [Implicit Bias comment] to be flagged up (in the debrief) as explicitly inappropriate rather than brushed under a carpet”
SBE facilitating a transformative learning experience	Participant 1: “It definitely challenged me to think a bit more deeply about the impact of the things we say about patients... I find myself also guilty of doing this from time to time, making jokes at the patients’ expense, when they’re not there” Participant 2: “I’ve got this kind of idea of the way I’d like to treat people but I don’t, um, I don’t live up to that it turns out. So that’s useful to identify”
Professional expectations of doctors to treat all patients fairly	Participant 2: “You hope that, you know being a doctor and looking after someone, you gain their full respect, but there are still some biases that really get in the way of that” Participant 2: “I’d like to think that I’m the kind of person who wouldn’t treat someone differently, if they were an alcoholic, or drug user, or whatever” Participant 3: “It’s kind of unexpected for... a registrar to have these sort of like prejudices... maybe not unexpected, but it shouldn’t be coming from them... It’s unprofessional.”
Challenges of confronting and dealing with microaggressions as a medical student	Participant 1 “But I guess that kind of shows that this is how I've always, you know, dealt with these sorts of incidents. Whether it's big or small, whether it's just a passing comment or whether it's a full-on confrontation…There's no code of conduct. Participant 4: “I do find it hard as a medical student because you’re very much like the bottom of the pile like you just feel it’s not your place to kind of, say anything or there’s someone else that should be saying something... I never want to like step on anyone’s toes... I feel there’s like quite a hierarchy and we often just have to like stay quietly in the corner and do what we’re told”

## O4. Co-designing simulation-enhanced training for first responder families

### Michelle O’Toole, Angeline Traynor, Anna Tjin, Brian Doyle, Claire Mulhall, Walter Eppich

#### The Royal College of Surgeons in Ireland

*Adv Simul 2023*, **8(1)**:O4

***Introduction***

First responders experience duty-related trauma exposures and thus more mental health problems compared with the general population. Little education and support exists for loved ones who first responders rely on for support. We describe the co-design of a simulation-enhanced programme which aims to: (1) explore first responder families' experiences of social support and (2) involve key stakeholders in programme development, and (3) to prepare first responder families to apply principles of psychological first aid.

***Methods***

This project involves several key collaborators to translate the collective insights of key stakeholders into the design of a simulation-based intervention: (a) RCSI SIM, an academic unit, (b) Mental Health Ireland, a charitable organisation, and (c) community partners including Dublin Civil Defence. Kern’s 6-step approach to curriculum development served as a guiding framework, supporting intervention development and delivery. Data collection included field observations, focus groups and semi-structured interviews during 6 co-design workshops. Thematic analyses were performed and shared with participants for further feedback. This research was approved by the RCSI Research Ethics Committee.

***Results & Discussion***

29 participants (16 first responders, 7 family members and 6 organisational representatives) shared experiences of critical incidents, help-seeking barriers and facilitators, and support preferences. Key themes identified the main barriers to help-seeking as: cultural stigma, ineffective communication, and perceptions of (tokenistic) organisational support.

Data also revealed gaps in participant support such as the need to share information with family members and the practical communication skills that help manage responses to critical incidents. We describe the co-design process using an example of how to engage first responders and their families effectively to co-develop and implement an innovative training programme. Using participatory research methods, we contribute to current understanding of the experiences of first responder families and their existing social support sources. We demonstrate innovation in the combined use of co-design and simulation to develop a practical intervention for first responder families. Our work contributes to the growing field of co-designed interventions.

***Keywords***

Curriculum development, simulation, mental health, co-design, first responders,

***Reference***

1. Thomas, P.A. & Kern, D. & Hughes, M.T. & Chen, B.Y.. (2015). Curriculum development for medical education: A six-step approach. Johns Hopkins University Press.

## O5. Cognitive interviews: a novel approach to post-event debriefing?

### Matthew B. Weinger^1^, Shilo Anders^2^, Laura Militello^3^, Arna Banerjee^2^, Amanda Burden^4^

#### ^1^Hospital virtual Valdecilla; ^2^Vanderbilt University Medical Center; ^3^Applied Decision Science; ^4^Cooper School of Medicine of Rowan University

*Adv Simul 2023*, **8(1)**:O5

***Introduction***

Might cognitive task analysis (CTA) methods conducted by non-clinicians have educational value during high-fidelity simulation training of clinicians? We explored this question during a multicenter study aimed at better understanding clinician decision-making during acute crisis events. We evaluated whether non-clinician-facilitated cognitive interviews immediately following simulation scenarios were deemed by experienced clinicians to be an effective method of self-reflection and learning.

***Methods***

Board-certified anesthesiologists (BCAs) managed four standardized simulated crisis events (postoperative hypotension, preoperative chest pain, postoperative dyspnea, & post-emergence agitation) during an all-day study. Immediately after each scenario, we conducted a 40-minute cognitive interview (CI) to ascertain ‘why’ and ‘how’ the participant made decisions during the scenario. Each participant was interviewed four times during the study day – there was no traditional debriefing or performance feedback until the very end of the study day. Instead, after each scenario, CIs, based on established CTA methods [1-2], were conducted by trained non-clinician interviewers. At the start of each interview, participants were asked to write down 3-5 of the most salient events that occurred during the scenario. The interviews were designed to allow participants to reflect on their thoughts, key assessments, contextual factors, decisions made, and actions taken. To evaluate the educational value, after the fourth CI (and prior to an end-of-day clinician-facilitated debriefing), participants completed a 7-item survey (5-level Likert scale) and two open-ended questions (Table 1A). Only then did participants receive a 30- to 60-minute traditional expert clinician-facilitated educational debriefing covering the entire day. To receive continuing medical education credit, participants responded to a course evaluation form sent electronically two days after the course by a separate organization (Table 1B).

***Results & Discussion***

As can be seen from Table 1A, the 42 participants reported very high agreement about the educational value of the CIs including for relevance, reflection, and psychological safety. This was supported by numerous narrative comments.

Scores on the CI survey were essentially indistinguishable from those in the traditional post-course survey completed by the same participants (Table 1B). These ratings are only slight lower than seen on similar post-course questions after traditional simulation-based maintenance of certification simulation courses for the same population [3 and 2021 unpublished]. These preliminary results suggest that cognitive interviewing could be an effective complementary approach to facilitating reflection and learning during high-fidelity simulation. Future studies should compare current debriefing paradigms [4] with CI-based debriefing of events in a more controlled experimental design.

***Keywords***

Debriefing, Interview methods, Cognitive task analysis, Cognitive engineering, Self-reflection, Learning

This work was funded by a grant from the Agency for Healthcare Research and Quality (AHRQ, R18-HS026158) to Vanderbilt University Medical Center (MB Weinger, PI).

***References***

1. Militello LG, Hutton RJB: Applied cognitive task analysis (ACTA): A practitioner's toolkit for understanding cognitive task demands. Ergonomics 41: 1618-41, 1998.
2. Hoffman R, Militello LG: Perspective on Cognitive Task Analysis: Historical Origins and Modern Communities of Practice. New York, NY, Taylor and Francis, 2008.
3. McIvor W, Burden A, Weinger MB, Steadman R: Simulation for maintenance of certification in Anesthesiology: The first two years. J Contin Educ Health Prof Sep 32(4): 236-42, 2012.
4. Lyons R, Lazzara EH, Benishek LE, Zajac S, Gregory M, Sonesh SC, Salas E: Enhancing the effectiveness of team debriefings in medical simulation: More best practices. Jt Comm J Qual Patient Saf 41(3): 115-25, 2015.

**Table 1 (Abstract O5):** Survey questions and responses

**A. Post-CI Survey Questions** *(n=42 … administered prior to any traditional debriefing)*	**% strongly agree or agree**
Today’s cases were relevant to my practice	92.9%
In my practice, I spend time after unusual clinical events reflecting (by myself or with others) on what might have been done differently^a^	90.4%
The cognitive interviewers helped me to reflect upon the decisions I made during the simulated clinical events.	97.7%
I enjoyed the cognitive interview process	90.5%
I felt psychologically safe enough to be candid during the cognitive interviews	100.0%
What I learned as a result of the cognitive interviews may change my practice.	83.2%
A cognitive interview like what you did today would be useful after actual critical events.	95.1%
**B. Post-course Evaluation Survey Questions** *(n=25 responding … sent by the ASA 2 days after course)*	**% strongly agree or agree**
This experience met its stated objectives	96%
The course content was relevant to my practice	90%
The experience was nonthreatening and conducive to learning	98%
The simulated clinical situations were realistic	94%
The program faculty and staff were effective in facilitating my learning	95%
I had an opportunity to reflect on my performance	100%
This was an effective use of my time	95%
The format was an effective way to learn this material	96%
Overall, this program was a positive learning experience	95%

^a^Scale was always/usually/occasionally sometimes. All other questions used the scale strongly agree/agree/undecided/disagree/strongly disagree

## O6. Decision-making and cognitive error: a novel undergraduate simulation session

### Amrita Brara, Julie Mardon, Beth Harrison

#### Scottish Centre for Simulation and Clinical Human Factors

*Adv Simul 2023*, **8(1)**:O6

***Introduction***

Cognitive errors occur when an individual has the necessary knowledge required to reach the correct solution, however their decision-making process is faulty. Indeed, cognitive factors have been repeatedly identified as having a key and preventable role in medical errors. Despite this, education around decision-making and its role in medical errors is lacking in medical undergraduate curricula in the UK.

Medical errors affect both patients and healthcare professionals. Increasing and necessary attention is now being paid to the psychological impact on the ‘second victim’, that is the person who is responsible for the mistake. This psychological toll can take the form of depression, anxiety, and suicidal thoughts. Emotional candour - being open and honest about your emotions following an experience - has been found to help healthcare professionals cope with errors in a more positive manner.

We designed a half-day undergraduate simulation session on decision-making and cognitive error.

***Methods***

Educational Objectives:

1. Describe the key theories related to decision-making
2. Compare and contrast System 1 and System 2 thinking
3. Explain the role of decision-making in cognitive error
4. Discuss the psychological impact of error on healthcare professionals
5. Analyse own and others’ decision-making process Learning Strategies:
  Icebreaker- Bomb defusal game
  Decision-Making Tutorial- dual process theory, decision-making in cognitive error
  Small-group Discussion- emotional candour around personal experiences of error
  Non-medical Tactical Decision Game Simulation- ‘Stranded on a Desert Island’

Medical Triage Tabletop Simulation – Emergency Department Patient Triage Session Overview: This novel undergraduate session will give medical students an understanding of decision-making and cognitive error. They will explore different theories around decision-making, in particular dual process theory. Through the use of tabletop simulation, they will analyse their own and others’ decision-making process, including the use of System 1 and System 2 thinking. They will develop an awareness and understanding of the role decision-making has in cognitive error in healthcare. They will participate in an open and honest conversation about their emotional responses in personal experiences with error.

***Results & Discussion***

This session will not only teach medical students about the decision-making process and its role in medical error, but uses the powerful tool of simulation to allow students to analyse and explore their own decision-making process in a safe environment prior to entering clinical practice as working doctors.

Furthermore, we hope that emotional candour following errors will help students learn to cope with mistakes in a more psychologically safe way.

***Keywords***

decision making, error

***References***

1. Plews-Ogan M, May N, Owens J, Ardelt M, Shapiro J, Bell SK. Wisdom in Medicine: What Helps Physicians After a Medical Error? Academic Medicine. 2016; 91(2):233-241.
2. Stiegler MP, Tung A. Cognitive Processes in Anesthesiology Decision Making. Anesthesiology. 2014; 120:204–217.
3. Stiegler M, Goldhaber-Fiebert S. Understanding and Preventing Cognitive Errors in Healthcare. MedEdPORTAL. 2015;11.
4. Stiegler MP, Neelankavil JP, Canales C, Dhillon A. Cognitive errors detected in anaesthesiology: a literature review and pilot study. BJA: British Journal of Anaesthesia. 2012;108(2):229–235.

## O7. Does sharing mean better caring? Shared leadership in healthcare emergency teams - implications for team training

### Sarah Janssens, Robert Simon, Michael Beckmann, Stuart Marshall

#### Mater Mothers’ Hospital, Monash University, University of Queensland

*Adv Simul 2023*, **8(1)**: O7

***Introduction***

Shared leadership in teams is associated with improved team performance in a wide variety of settings but is under-researched in healthcare emergency teams. Leadership sharing exists in many trauma and emergency teams however impact on team performance is unknown. This research aimed to understand the relationship between both spontaneous and planned leadership sharing and team performance in simulated maternity emergencies.

***Methods***

Native teams attending a maternity emergency training day were the subject of two sequential studies. Initially an observational study calculated the distribution of leadership within the team using utterance coding and compared low and high leadership sharing teams. Data from this study informed a planned leadership sharing structure which was examined in a counterbalanced crossover trial. In the planned shared leadership scenarios, teams implemented a “logistics leaders” who was responsible for team and resource co-ordination and communications. The primary outcome measure in both studies was teamwork, as assessed by the Auckland Team behaviour tool. Secondary outcome measures included time to critical intervention, clinical checklist completion and in the planned leadership study, self-assessed teamwork (TEAM tool) and workload.

***Results & Discussion***

Sixteen teams were included in the observational study with universal spontaneous leadership sharing noted. High and low sharing teams had similar teamwork scores (5.02 vs 4.96, *p*= 0.574). Low sharing teams had faster time to critical intervention (193s vs 312 s *p*=0.018), but checklist completion was not different. Thirty-two teams participated in the planned shared leadership intervention, with no difference seen in the within-teams analysis of teamwork scores in the singular compared to planned shared leadership scenarios (5.3 vs 5.3, *p* = 0.91). Secondary outcomes of checklist completion, time to critical intervention, self-assessed teamwork and workload were all similar between the singular and planned shared leadership scenarios.

Objective data from these studies does not suggest a benefit of shared leadership within the maternity emergency team context. While the observational data suggested a benefit to a more concentrated leadership distribution, the planned shared leadership dyad studied prospectively did not result in improved team performance. Teams training in shared leadership models should consider the risks and benefits of shared leadership in each context, particularly the division of leadership tasks and how co-leaders work together most effectively. Teams training in a singular leadership model, should concentrate on how a singular leader can be supported by other leadership in the team that enhances, not distracts or disrupts from, their leadership.

***Keywords***

Maternity, Obstetrics, Leadership, Emergency teams

***Acknowledgements***

This abstract summarizes research contributing to my PhD obtained through Monash University.

***References***

1. Wu Q, Cormican K, Chen G. A meta-analysis of shared leadership: Antecedents, consequences, and moderators. Journal of Leadership & Organizational Studies 2020;27(1):49-64. 10.1177/1548051818820862.
2. Janssens S, Simon R, Beckmann M, et al. Shared Leadership in Healthcare Action Teams: A Systematic Review. J Patient Saf 2018 10.1097/pts.0000000000000503 [published Online First: 2018/06/06]
3. Janssens S, Simon R, Barwick S, et al. Leadership sharing in maternity emergency teams: a retrospective cohort study in simulation. BMJ simulation & technology enhanced learning 2020;6(3):135-39. 10.1136/bmjstel-2018-000409 [published Online First: 2020/04/20]
4. Janssens S, Simon R, Barwick S, et al. Midwifery leadership in maternity emergencies: a video analysis. Journal of Interprofessional Care 2019:1-7. 10.1080/13561820.2019.1675611.
5. Janssens S, Clipperton S, Simon R, et al. Clinicians’ attitudes towards a co-leadership structure for maternity emergency teams: An interview study. Journal of Interprofessional Care 2022;
6. Janssens S, Clipperton S, Simon R, et al. Coleadership in Maternity Teams, a Randomized, Counterbalanced, Crossover Trial in Simulation. Simulation in Healthcare 2022

## O8. Establishing a government mandated national collaborative network for simulation-based learning – what is next?

### Benedicte Skjold-Ødegaard^1^, Pål Andre Hegland^2^, Turi Hauan^3^, Stine Gundrosen^4^, Helle Madsen-Holm^5^, Sigrun Anna Qvindesland^1^, Liv Skinnes^5^, Gudmund Rørheim^1^, Une Stømer^1^

#### ^1^Stavanger University Hospital; ^2^Førde Hospital; ^3^UNN; ^4^St Olavs Hospital; ^5^Oslo University Hospital

*Adv Simul 2023*, **8(1)**:O8

***Introduction***

Norway is a scarcely populated and elongated country – and hospital-based simulationists have traditionally developed and performed their simulation-based learning (SBL) activities on their own. In 2020, the Norwegian government formally recognized SBL as an important factor for improved patient safety in healthcare – and highlighted the need to coordinate SBL nationally 1. This was the background for establishing InterRegSim - a government mandated national collaborative network for SBL in the Norwegian regional health trusts. InterRegSim was presented as “Hot topic” in SESAM 2022 Seville and this abstract is an update on the network’s subsequent work.

***Methods***

InterRegSim reports to the regional Chief Executive Officers (CEOs) in the four Norwegian regional health trusts. The initial mandate issued by the CEOs emphasized that the network should ensure a national structure and system for simulation and skills training, facilitate learning between health trusts and promote cooperation between different SBL environments.

After InterRegSim was established in January 2022, the group experienced the initial mandate to be somewhat diffuse. We therefore made it one of our first tasks to clarify the mandate. We structured our responsibilities into four main domains: Systematic evaluation, Competency development of healthcare workers using SBL, Professional development of SBL faculty, and Sharing (Figure 1). The clarification of the mandate will be presented to the CEOs during autumn 2022 to ensure the mandate is still in line with the task given to InterRegSim.

As an aim to facilitate cooperation and sharing among simulationists in Norway, we created a website 2, which serves as a platform for disseminating our work – and other relevant work - nationally as well as internationally. The website is intended to be a resource for simulationists, clinicians, leaders and other stakeholders. We are currently working on developing indicators that can be used to measure the impact of the collaborative simulation network.

***Results & Discussion***

A network is defined as “a large system consisting of many similar parts that are connected together to allow movement or communication between or along the parts, or between the parts and a control center” 3. This definition works well with our mandate and InterRegSim can be considered the “control center” for SBL in the Norwegian public hospital system. InterRegSim works to increase the use of SBL, to learn from each other and to share available knowledge – with increased patient safety as the desired outcome.

***Keywords***

N.A

***References***

1. Nasjonal helse- og sykehusplan 2020-2023 - regjeringen.no
2. InterRegSim - Helse Stavanger (helse-stavanger.no)
3. Cambridge Dictionary | English Dictionary, Translations & Thesaurus

**Fig. 1 (Abstract O8).**
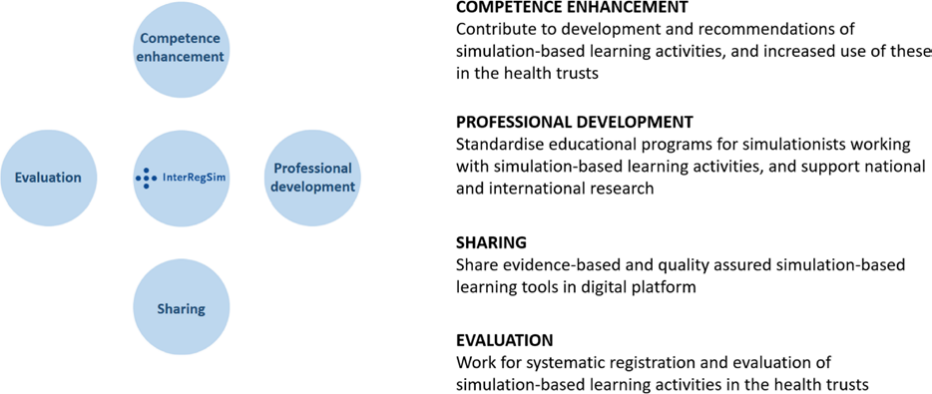
See text for description

## O9. Life-support training course development for the civilian population and medical staff during the ongoing war in Ukraine

### Olena Korotun^1^, Cristina Alvarado^2^, Kristopher R. Brickman^2^, Ruslan Knut^1^, Olena Nechytailo^1^, Oleksii Godovanets^1^, Iryna Kozlovska^1^, Vitalii Smandych^1^

#### ^1^Bukovinian State Medical University; ^2^University of Toledo

*Adv Simul 2023*, **8(1)**: O9

***Introduction***

War beginning in Ukraine become a huge emerging healthcare challenge and correspondingly a challenge for medical education and simulation. Within a short period, we had to completely change priorities in simulation training. Existing training for first emergency care and basic life support does not meet the needs of the population and medical staff when a constant threat of explosions and military traumas exists.

***Methods***

Centre of Simulation Medicine and Innovative Learning Technologies (COSMIT) of Bukovinian State Medical University (BSMU), Chernivtsi, Ukraine working since 2018, from the first week of the war started receiving many requests from the population, healthcare institutions, local authorities, and local defense force offices to conduct training on emergency care and life support in case of an explosion and military traumas. During the first 7 months of the war, we trained over 3000 people of different categories. The training program was constantly updated based on feedback and reports from the front line. Since April 2022 COSMIT team started cooperation with the Simulation Centre of the University of Toledo. Based on this cooperation an updated and adapted to the National Healthcare system training course for the “first on the scene” categories was developed and successfully conducted for the 400 police officers of the Chernivtsi region.

***Results & Discussion***

The simulation training culture in Ukraine is in the stage of fast development. The training in tactical medicine for officers became more and more relevant in recent years [1]. Military medicine is a unique niche of healthcare [2] and requires a multidimensional and multidisciplinary approach. Developing and implementing simulation training courses of emergency care in case of military trauma and explosions for different categories of trainees today is an extremely relevant task in Ukraine. However, it has many challenges as the absence of a unified approach to trauma care at all levels in Ukraine as the ATLS course approach [3]; supply deficiency and low-quality not certified or self-made supply and equipment; lack of experience in military and explosive trauma care in physicians; lack of experienced and certified trainers. The main further perspective for our Center is to develop an adapted and updated advanced military and explosive trauma life support course for Ukrainian physicians; develop a unified National approach to trauma support in the country.

***Keywords***

Emergency care, trauma life support

***References***

1. Maistrenko . ., Bubenshchykov . ., & Stetsiv . . (2020). The use of simulation modelling tools in a practical training for the prospective officers of the armed forces of Ukraine. Information Technologies and Learning Tools, 75(1), 186–201. 10.33407/itlt.v75i1.2676.
2. CPT Jessica Eker, CPT Hugh Hiller, LTC (P) Guyon Hill, et al. Preparing Emergency Physicians for the Next War: Residency Capstone Training in Prolonged Casualty Care. p.10-17. The Medical Journal, April-June 2022, p.10-17.
3. Abu-Zidan FM. Advanced trauma life support training: How useful it is? World J Crit Care Med. 2016 Feb 4;5(1):12-6. 10.5492/wjccm.v5.i1.12. PMID: 26855889; PMCID: PMC4733451.

## O10. Multiprofessional simulation training to prevent the risk of clinical deterioration and to improve teamwork and communication, supported by augmented intelligence: the vigilance project

### Esther León Castelao^1^, Munt Garcia Font^1^, Pedro Cartaxo Cintra^2^, Pedro Castro Rebollo^2^, Miquel Sanz Moncusí^2^, Nelson Gabriel López Esquivel^1^, Miguel Fernández Santana^1^, Rocío Ponce Muñoz^1^, Iago Enjo Perez^1^, José María Nicolás Arfelis^2^

#### ^1^Universitat de Barcelona; ^2^Hospital Clínic de Barcelona

*Adv Simul 2023*, **8(1)**:O10

***Introduction***

Regarding the latest evidence that remarks the risk of a higher morbimortality rate due to the lack of a standardised screening and escalation guidance of acute patients admitted in the hospital wards. The Hospital Clínic of Barcelona considered to developed a transversal project that aims to improve the detection and response of patients’ clinical deterioration – Vigilance Project (VP).

A patient surveillance system supported by augmented intelligence was designed – ClinScreen – in order to automatically measure different scoring systems as NEWS2 . It also assists healthcare professionals (HP) to monitor and analyse certain clinical parameters from the electronic patient records and provides clinical information that guides escalation. VP empowers each HP to escalate according to the clinical judgement and the ClinScreen analysis results, which entails the need of effective teamwork and communication skills. Considering the novelty of the two main axes, the project’s development team identified the need to design a simulation-based multiprofessional educational program to prepare the HP for its implementation.

***Methods***

The educational program was divided into two phases: 7hours – e-learning approach; 7hours – simulation-based approach. The e-learning phase prompts the acknowledgment of the project, their characteristics, the tools and procedures with videos of experts and interactive activities – knows and knows how. Subsequently, the simulation-based approach is entitled to practise the technical and non-technical skills addressed in the previous phase with clinical cases – demonstration of learning.

A total of 500 HP from 16 wards (309 beds) with different roles/specialities were invited to join the first edition of the educational program.

The simulation-based approach includes the initial enrolment of three SimZone 1 scenarios: SBAR and multiprofessional communication; ClinScreen and NEWS2 ; ABCDE clinical assessment. Considering the objectives of these simulation scenarios, rapid cycle deliberate-practice has been elected as the main instructional strategy. Afterwards, three clinical case-scenarios were designed (SimZone 2) to simultaneously apply the contents previously developed: a dynamic plus/delta educational approach was adopted.

***Results & Discussion***

The simulation training facilitates the implementation of a complex and novel patient surveillance system that might have a significant impact in the clinical safety and quality of care. Feedback was received and analysed from the HP and supports the opportunity to adjust and improve the protocol with their recommendations.

Nevertheless, through this simulation-based approach, there is a new possibility to assess the actual needs of the HP in the clinical setting, promoting the analysis of active safety and educational gaps that the institution might need to consider.

***Keywords***

Multiprofessional education; Teamwork; Communication; Clinical deterioration; Augmented Intelligence

***References***

1. DeVita, M.; Hillman, K.; Bellomo, R. Textbook ofRapid Response Systems, 2nd ed.; DeVita, M.A., Hillman, K., Bellomo, R., Odell, M., Jones, D.A., Winters, B.D., Lighthall, G.K., Eds.; Springer International Publishing: Berlin/Heidelberg, Germany, 2017.
2. Mitchell, O.J.L.; Motschwiller, C.W.; Horowitz, J.M.; Evans, L.E.; Mukherjee, V. Characterising Variation in Composition and Activation Criteria of Rapid Response and Cardiac Arrest Teams: A Survey of Medicare Participating Hospitals in Five American States. BMJ Open 2019, 9, e024548.
3. Roussin CJ, Weinstock P. SimZones. Acad Med [Internet]. 2017 Aug;92(8):1114–20. Available from: http://journals.lww.com/00001888-201708000-00029

## O11. NeoSim: a multiprofessional clinical education, quality improvement initiative

### Sarah Berry, Maureen O'Dowd, Aoife McMorrow, Diane Chalkright

#### Belfast Health and Social Care Trust

*Adv Simul 2023*, **8(1)**:O11

***Introduction***

The COVID-19 pandemic has obliterated all signs of normality in healthcare and medical education, forcing Educators to review the delivery of teaching. Awareness of this prompted the development of a new learning platform that is accessible and relevant to all members of the Neonatal Multidisciplinary team (MDT).

Aims:

- Create a collaborative learning network to promote shared learning from an interprofessional MDT.
- Use simple technology to enhance learning through an easily accessible, inclusive, and socially distanced format.
- Increase diversity and inclusion of clinical education within the MDT.
- Enhance knowledge and understanding of other professional roles and aims in neonatal care, promoting a holistic approach to patient care.

***Methods***

The significant benefits of collaborative working and shared learning is not new philosophy, but a well proven fact. The delivery of interprofessional training leads to improved team-working by facilitating a better understanding of other team members perspectives, alongside increasing staff motivation and morale.

An awareness of the importance of collaborative learning led to the creation of Neosim, an interprofessional, educational team. The aim of the group was to create a bank of evidence based, quality assured and peer reviewed educational videos. We began by surveying all members of the team for their most desired topics, to ensure our content was relevant to staff need. The categories ranged from practical procedures and equipment tutorials to disease management and guideline reviews. A YouTube channel was then created to make the content easily accessible to the neonatal network across Northern Ireland.

***Results & Discussion***

The educational videos are a unique, fun, and informative resource that anyone with a neonatal interest can access at any time, from any location. The videos have also been used to aid regional teaching, practical courses and medical and nursing staff inductions, with excellent feedback received. Over 30 videos have been created to date, with more in production and the list of future topics continuing to grow. The project would not be possible without the fantastic contribution from our nursing, midwifery, physiotherapy, occupational therapy, dietetics, pharmacology, critical care technologists and medical teams. Following their creation, the videos are reviewed and published by our local MDT. Through helpful feedback and ongoing submission of ideas, future clips will be created using a PDSA cycle. Moving forward we plan to increase our collaboration of shared learning regionally amongst trusts in Northern Ireland and nationally with other paediatric schools in the UK.

***Keywords***

Neonatology, Medical Education

***Reference***

1. Bernard O'Donnell, BHSCT videographer.

## O12. Paramedicine students as simulated patients in an introduction program for first year residents (FY1)

### Hilde Klippen Hetland^1^, Nina Vatland^2^, Jorunn Flaten Lyngset^3^

#### ^1^SAFER, ^2^Stavanger University, ^3^Stavanger University Hospital

*Adv Simul 2023*, **8(1)**:O12

***Introduction***

First year residents (FY1) at Stavanger University Hospital (SUH) attend a mandatory introduction program before starting their work at the hospital. The FY1s have provided feedback that they wanted more scenario-based simulation to help them prepare to master clinical challenges at the hospital. Through collaborative work between the SUH and the bachelor program for paramedicine at the University of Stavanger, we discovered an opportunity to improve learning and realism in the scenarios, provide interprofessional learning opportunities, and achieve academic and residential learning goals. Our pilot work focused on preparing and safely involving 2nd year paramedic students as simulated patients in these scenarios.

***Methods***

The paramedicine students entered the role of simulated patients during these days and achieved two curricular learning goals:

1. Have broad knowledge of methods for patient examination, history taking, assessment and measures related to injuries or diseases in one or more organ system
2. Can reflect on and have insight into the patient`s perspective

Student preparation methods were undertaken in accordance with The Association of Standardized Patients Educators (ASPE) (1). The students attended a half-day preparation course before the introduction program including opportunities to get acquainted with scenarios (12), chosing which scenarios to participate in, peer-to-peer training with instructor supervision and feedback on simulated patient behaviour, empowering students to collaborate with physician facilitators and focusing on the patients’ perspective in the debriefing. Two simulation experts from the hospital coached the students how to present patient cases realistically.

***Results & Discussion***

Involvement of the paramedic students in this introduction program was well evaluated by both the facilitators (experienced doctors), FY1s and the students. Post-course feedback from the students was collected. Some examples were: “I learned: how to speak respectfully and understandably to the patient; how to treat COPD exacerbation; the importance of patience and respect, especially in psychiatry. The students experienced being simulated patients as valuable learning. They had suggestions for improvement including desire for more preparation time, disappointment in only being able to participate as a simulated patient in one case instead of having the opportunity to be involved in several. We will use these suggestions to improve the next introduction program for FY1. The paramedicine students contributed to realism in the scenarios, experienced interprofessional learning and achieved curriculum objectives.

Interprofessional simulation in education, introduction program for residents, simulated patients, paramedicine

***Reference***

1. Lewis K. L., Bohnert C. A., Gammon W. L., Holzer H., Smith C., Thompson T. M. & Gliva-McConvey G. (2017). The Association of Standardized Patient Educators (ASPE) Standards of Best Practice (SOBP). Advances in Simulation 2:10. 10.1186/s410.

## O13. Preparation for practice - interprofessional simulation based education

### Kath Sharp, Elizabeth Simpson, Stephen Patterson, Ciara King, Neil MGowan

#### NHSGGC

*Adv Simul 2023*, **8(1)**:O13

***Introduction***

There is international agreement that pre-registration healthcare students should experience inter-professional education (IPE) to prepare them for practice [1]. Within the United Kingdom, Higher Education Institutions (HEI) are embedding IPE as part of pre-registration curriculums. However, due to the complexity of managing multiple curriculums from multiple institutions as well as increased resource allocation requirements, delivering IPE through simulation-based education (SBE) to pre-registration health care students remains a challenge. We developed, implemented and evaluated an inter-professional clinical SBE course delivered to pre-registration medical nursing and pharmacy students as they prepared to join the work force. Research aims included exploring the participants and faculties experience of participating in the course.

***Methods***

A group of inter-professional simulation educators from three HEIs in the West of Scotland worked collaboratively to develop the intended learning outcomes (ILO) and design the SBE course. During the course up to six students (three medical, two nursing and one pharmacy) worked together in a simulated medical ward scenario to prioritise and deliver care to patients. A mixed methods approach was used to evaluate the course which included an online survey completed by participants immediately following the course. The participants will be invited to participate in focus groups approximately 6 months after they have joined the workforce to explore their experience of IPE in preparing them to practice. In addition, the faculty were invited to participate in focus groups to explore their beliefs around IPE and their experience of facilitating IPE.

***Results & Discussion***

Preliminary data from the online survey is presented here. A total of 65 courses were delivered by 105 faculty to 232 student participants (178 medical, 33 nursing and 21 pharmacy). 137 students (115 medical, 19 nurses, 13 pharmacy) completed the online survey. Thematic analysis was used to code the text responses that were of interest and relevance to the research aims and the ILOs. The majority of participants valued the opportunity to learn alongside other professionals to enhance their communication skills. Many of the participants discussed that they had developed a better understanding of the importance of working with other professionals to deliver better patient care. Many of the participants discussed the benefits of having an opportunity to consider their abilities and understanding the abilities of other professionals. The findings of the online survey suggest that some of the ILOs were met. To deepen our understanding, further exploration will take place in the focus groups.

***Keywords***

Interprofressional Education undergraduate

***References***

1. World Health Organisation (2010) Framework for action on interprofessional education and collaborative practice. https://www.who.int/news-room/fact-sheets/detail/nursing-and-midwifery.
2. Centre for Advancement of Interprofessional Education (2021) Interprofessional Handbook. www.caipe.org.

## O14. SimInPath: mobile application to assess skills in pathology

### Eduardo Alcaraz-Mateos^1^, Kristina Ilic^2^, Aliz Kovács^3^, Fuensanta Caballero-Aleman^1^, Manuel Párraga-Ramírez^1^, Nicolas Sanchez-Campoy^4^, Enrique Poblet^5^

#### ^1^Morales Meseguer University Hospital, UCAM; ^2^Faculty of Medicine, University of Belgrade; ^3^Faculty of Medicine, University of Pecs; ^4^National Statistics Institute; ^5^Reina Sofia University Hospital and Faculty of Medicine, University of Murcia

*Adv Simul 2023*, **8(1)**:O14

***Introduction***

As an academic subject, Pathology is eminently theory based, and is routinely assessed in this fashion. The objective of this project was to design a nonprofit application for mobile devices to assess competencies or practical skills in Pathology.

***Methods***

There was an initial phase of development for the application and a second phase for testing. A template for evaluation was created and included the most relevant descriptors for each stage for the following skills: macroscopic dissection, palpation of lesions suitable for fine needle aspiration (FNA), FNA, and ultrasound-guided FNA (USFNA). For every module, each item could be evaluated using a Likert scale checklist. A free-text field was also created to allow evaluators to include additional observations, while times for completing the different modules were registered. During the testing and evaluation phase, two evaluators (a pathologist and a nurse, histotechnician in training) assessed performance of a group of international medical students (*n*=7), after previous training in the skills with flipped classroom and simulation-based methodology. Inter-rater concordance analysis was performed using the kappa coefficient.

***Results & Discussion***

A private company was hired to develop the SimInPath® (Simulation in Pathology) application, which took 8 weeks to complete. The application became available for download through the GooglePlay (Android) and App Store (iOS). It was determined that the nonprofit application would be free to download, dispensing with advertising content.

- Students completed the training and were evaluated, obtaining scores between 8,5 and 9.2 out of 10 (average rating of two evaluators). The overall inter-rater agreement between faculty was substantial (k=0,752 *p*<0.001), being perfect for the dissection module (k=1), followed by the USFNA (k=0,752), the FNA (k=0,748), and the palpation module (k=0,508). Conclusions: The SimInPath® app for mobile devices to assess practical skills in Pathology was designed and launched.
- This tool could be used to implement simulation-based medical education in medical school or during residency, and, indeed, it could be used in the objective structured clinical examination (OSCE) assessment formats. In addition, due to the agreement between evaluators found, different professionals could act as evaluators.
- In addition, it could provide greater visibility of pathologists’ work and increase students’ interest in Pathology.

***Keywords***

App; Skills; Assessment; OSCE; Evaluation; Pathology

**Fig. 1 (Abstract O14).**
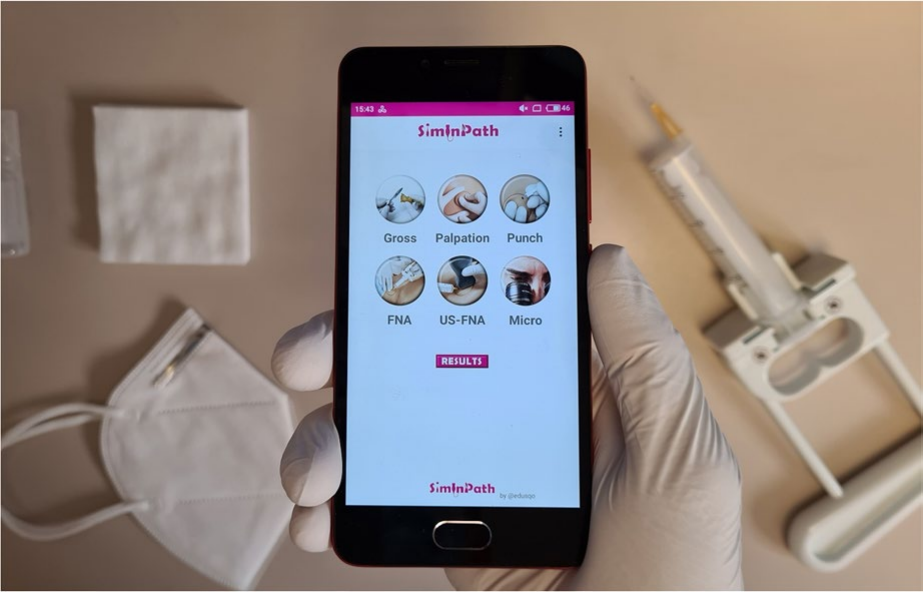
See text for description

## O15. SimLab: an ethnographic case study of a cultural historical activity theory formative intervention to improve emergency care preparedness in general practice

### Sarah O’Hare^1^, Anu Kajamaa^2^, Gerard Gormley^1^

#### ^1^Queens University Belfast; ^2^University of Oulu

*Adv Simul 2023*, **8(1)**:O15

***Introduction***

The critical nature of emergencies requires General Practice (GP) practices to provide a prompt patient-centered collaborative approach. However, emergencies do not happen frequently enough for staff to gain and maintain such competencies.

In-situ simulation (ISS), simulation training in working clinical environments, is documented as an acceptable and feasible way to train for GP emergencies. However, further studies are required to explore whether ISS can result in practice change(1).

Little research attention has been directed to the role of theoretical models in facilitating a collective creation of knowledge and learning with ISS(2). Cultural Historical Activity Theory (CHAT) is a useful methodological framework to study practice-based learning in complex learning environments(3). The lens of CHAT was applied to an ethnographic case study to gain a deeper understanding of organizational change brought about by an ISS training model of emergencies.

***Methods***

Change Laboratory (CL) is a formative intervention method developed for studying workplaces in transition and generating improved, shared patterns of activity(4). A smaller scale adaptation of a CL, ‘SimLab’ was developed for the busy GP workplace.

An interprofessional team participated in the ‘SimLab’ which included four workshops (Figure 1). Workshop (I) involved a ‘mocked-up’ paediatric ISS. Afterwards, participants were provided with a summary of recognised best practice, researchers facilitated participants in mapping workplace activity to CHAT framework and identified contradictions. In Workshop (II), through collaborative activity participants identified accumulated historical tensions and devised a future model to resolve contradictions(5). Workshop (III) involved a different ISS followed by a discussion to explore if the changes had enhanced their emergency response. During the fourth workshop the team discussed if the elements of their newly modelled system had become established. Qualitative data collection methods included focus group recordings, video footage and participant reflection diaries.

***Results & Discussion***

CHAT enabled a deeper exploration of the complex relationship of ISS and real-world clinical practice(6), enabling participants to take a systemic perspective by examining rules, division of labour, and tools that mediate workplace learning(7). Between activity system elements challenges occurred, reframed as opportunities, they enabled contradictions to act as potential levers for effective organisational change. The use of ISS provided real life contextuality, identified latent threats and has the potential to transfer well into practice enhancing the degree of preparedness for such time-critical, low-frequency, and high-morbidity events. This research demonstrates how CHAT can provide a theoretical lens and approach to guide ISS in healthcare(2) to bring about organizational transformation for emergency care.

***Keywords***

General Practice

***References***

1. Kalidindi S, 1 MK, Elliot Griffith 3. In-Situ Simulation Enhances Emergency Preparedness in Pediatric Care Practices. Official Journal of the American Academy of Paediatrics. 2018;142(1).
2. Gormley GJ, Kajamaa A, Conn RL, O’Hare S. Making the invisible visible: a place for utilizing activity theory within in situ simulation to drive healthcare organizational development? Advances in Simulation. 2020;5(1).
3. Qureshi SP. Cultural Historical Activity Theory for Studying Practice-Based Learning and Change in Medical Education. Advances in Medical Education and Practice. 2021;Volume 12:923-35.
4. Engeström Y, Pyörälä E. Using activity theory to transform medical work and learning. Medical Teacher. 2021;43(1):7-13.
5. Morris C, Reid A-M, Ledger A, Teodorczuk A. Expansive learning in medical education: Putting Change Laboratory to work. Medical Teacher. 2021;43(1):38-43.
6. Bearman M, Greenhill J, Nestel D. The power of simulation: a large scale narrative analysis of learners’ experiences. Medical Education. 2019;53(4):369-79.
7. Meijer LJ, De Groot E, Honing-De Lange G, Kearney G, Schellevis FG, Damoiseaux RAMJ. Transcending boundaries for collaborative patient care. Medical Teacher. 2021;43(1):27-31.

**Fig. 1 (Abstract O15).**
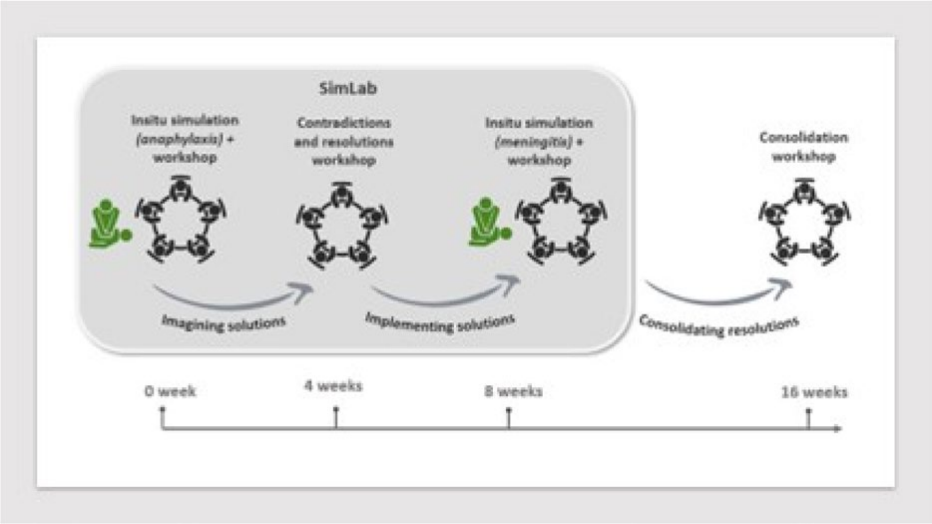
See text for description

## O16. Simulation based learning designed with a systems approach to enhance induction for general practice trainees.

### Joanna Traynor^1^, Sarah Luty^2^, Duncan McNab^2^, Sharon Donaghy^1^

#### ^1^NHS Lanarkshire; ^2^NHS Education for Scotland

*Adv Simul 2023*, **8(1)**:O16

***Introduction***

Many trainees commencing General Practice (GP) training have little experience of GP systems due to completing foundation training in hospital settings. Systems in GP vary with different policies, protocols and processes. GP trainees need an induction programme to orientate them to their new work setting. There has been little face-to-face training during the coronavirus pandemic reducing peer support and locally trainees reported high levels of isolation and low morale. A ‘GP emergencies’ simulation course was developed, delivered and evaluated to support trainee induction and improve trainee wellbeing.

***Methods***

Four scenarios were designed using the Systems Engineering Initiative for Patient Safety (SEIPS) framework to highlight important system factors relevant to work in a GP setting. One scenario (patient with hypoglycaemic episode) focused on practice organisation factors. A second scenario (baby with meningococcal septicaemia) on tasks such as medicine administration. The learning outcomes of the third scenario (patient with anaphylaxis) related to technology and tools.

The fourth scenario (a home visit with opiate overdose) explored environmental factors. Debriefs highlighted interactions between different system factors using the SEIPS framework.

Scenarios were adapted to reflect cultural events such as Ramadan, and the meningococcal rash was adapted for various skin types.

Evaluation immediately following and at six weeks after the session explored changes in knowledge, skills, confidence, and behaviours that may increase patient safety. In addition, impact on practitioner wellbeing and morale were assessed.

***Results & Discussion***

Trainees reported benefits from this course as part of their induction to practice as it highlighted key difference between primary and secondary care. They reported improved confidence and knowledge recognising and responding to emergencies and improved skills using equipment in a GP setting. They described adapting systems in their own practice such as creating emergency equipment checklists. Although an improvement in wellbeing was not noted, improved morale from sharing experience was reported. Reported reduction in feelings of isolation was noted.

A learning need for identifying skin rashes on patients with darker skin was identified. Review of the literature and educational resources revealed difficulty identifying pictures of this rash on such patients. Learning resources can be developed.

Future work will develop scenarios to support learning in other areas to which GP trainees have less exposure. This includes emergency scenarios with communication barriers, and psychiatric emergences in primary care, which specifically highlights learning on complex interacting factors within primary care systems.

***Keywords***

Systems Approach, General Practice, Emergencies, collaboration

***Acknowledgements***

Thanks to Neil McGowan, Associate Director Medical Education (Simulation) Greater Glasgow and Clyde NHS

## O17. Simulation-based mastery learning training curriculum for ultrasound guided interventional radiology procedures

### Andrea Doyle^1^, Claire Condron^1^, Coilin Cantwell^2^, Richard Arnett^1^, Claire Mulhall^1^, Walter Eppich^1^

#### ^1^RCSI University of Medicine and Health Sciences; ^2^St. Vincent’s Private Hospital

*Adv Simul 2023*, **8(1)**:O17

***Introduction***

Interventional radiology (IR) is an efficacious, safe and minimally invasive alternative to conventional surgery. The success of IR procedures rely on interventional radiologists’ ability to interpret diagnostic images to guide and manipulate surgical apparatus in the body through small surgical incisions. IR training currently follows the apprenticeship model, not linked to a robust curricular framework and requires significant resources, including mentored training time, longer operative times, and the potential harm to patients. Simulation-based medical education (SBME) successfully contributes to quality health professions education1, and is an effective educational model in improving IR trainee competency2. SBME provides the learner with the opportunity to practically implement their IR knowledge through the use of physical simulators; where the stakes are lower than those in the surgical environment and there is the opportunity to fail-safely while ensuring patient safety3.

***Methods***

This work describes the development of a simulation-based training curriculum for ultrasound guided IR procedures through Simulation Based Mastery Learning (SBML). This approach incorporates anatomically correct interactive simulation models at various stages of complexity. To design the curriculum and the training devices, the critical performance steps of IR kidney procedures were identified through cognitive task analysis (CTA) and a Modified Delphi Process consensus building activity illustrated in figure 1. Mastery learning is a competency-based approach to learning. It is learner centred and focuses on the achievement of a standard without curricular time constraints; learners acquire, competencies, measured and compared with fixed achievement standards, without limitations on time 4. There are seven features to mastery learning4, and the fourth feature relates to standard setting and is an essential facet of mastery learning. Standard setting should be rigorous and evidence informed. Mastery Angoff standard setting method5 employed in this study determined the minimum passing standard (MPS). The Mastery Angoff technique is also a consensus process that requires experts to judge the percent of learners who would achieve a threshold score on a checklist of items, and can do the procedure safely and independently for patient care6.

***Results & Discussion***

The curriculum was designed around the critical competencies that were identified from the Modified Delphi/CTA process. Commencing curriculum design while the IR experts engaged in the consensus process and the standard setting activities provided affordances and enabled an ongoing dialogue between the IR experts and the research team, thus enabling refinement during the evolution of the curriculum.

***Keywords***

Simulation-Based Mastery Learning, Health Professions Education, Modified Delphi, Cognitive Task Analysis, Interventional Radiology, Ultrasound Image Guidance, Simulator Training Device

***Acknowledgements***

The first author would like to acknowledge funding support from the Irish Research Council Government of Ireland Postdoctoral Fellowship Programme and the RCSI Office of Research and Innovation.

RCSI SIM is a CAE Healthcare Centre of Excellence and receives unrestricted funding to support its educational and research activities.

***References***

1. McGaghie, W. C., Issenberg, S. B., Petrusa, E. R. & Scalese, R. J. Revisiting ‘A critical review of simulation-based medical education research: 2003-2009’. Med. Educ. 50, 986–991 (2016).
2. Patel, R. & Dennick, R. Simulation based teaching in interventional radiology training: is it effective? Clin. Radiol. 72, 266.e7-266.e14 (2017).
3. Kneebone, R. Simulation in surgical training: educational issues and practical implications. Med. Educ. 37, 267–277 (2003).
4. McGaghie, W. C. Mastery learning: It is time for medical education to join the 21st century. Acad. Med. 90, 1438–1441 (2015).
5. McGaghie, W. C., Barsuk, J. H. & Wayne, D. B. Comprehensive Healthcare Simulation: Mastery Learning in Health Professions Education. (Springer International Publishing, 2020). 10.1007/978-3-030-34811-3.
6. Kane, M. Validating the Performance Standards Associated With Passing Scores. Rev. Educ. Res. 64, 425–461 (1994).

**Fig. 1 (Abstract O17).**
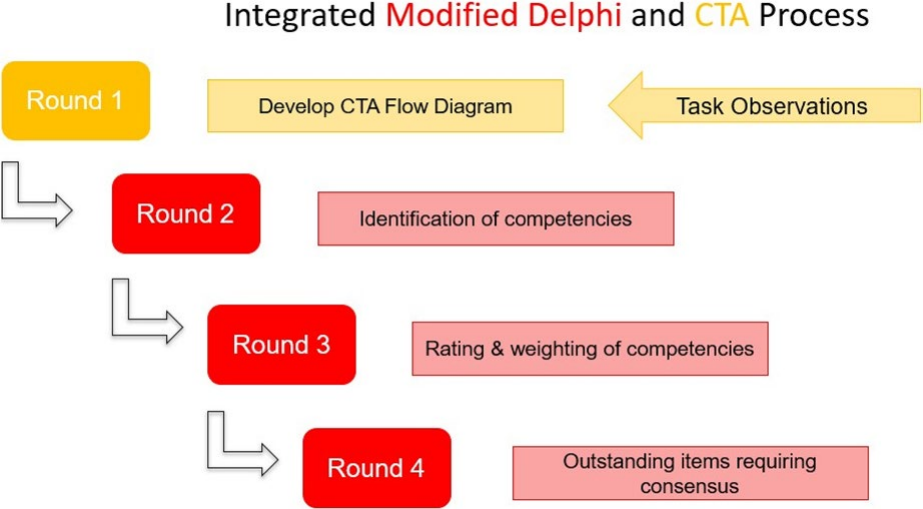
See text for description

## O18. Simulation-based training program for PICC insertion: randomized comparative study of synchronous direct feedback versus asynchronous distance feedback

### Pablo Besa, Gaspar Ramírez, Eduardo Abbott, Fernando Altermatt, Marcia Corvetto, Eduardo Kattan, Víctor Contreras, Elga Zamorano

#### Pontificia Universidad Católica de Chile

*Adv Simul 2023*, **8(1)**:O18

***Introduction***

Simulation training with deliberate practice has shown to be effective for procedural skill training (1,2). To find more cost-efficient training, delivering feedback remotely and asynchronously has been explored (3). The effectiveness of this type of feedback has not been evaluated for peripherally inserted central catheters (PICC). The objective of this prospective randomized study is to compare 2 different feedback techniques for this procedure: Synchronous direct feedback versus asynchronous distance feedback.

***Methods***

Prior approval of the institutional ethics committee, 40 anesthesia and internal medicine residents were recruited. Residents reviewed the instructional material in an online platform and performed a pre-training assessment (PRE), which consisted of a PICC insertion in an ultrasound guided part task trainer.

Residents were randomized to 2 types of training:

- Practice with direct synchronous direct feedback (SYNC). The instructor was present and giving instructions in real time throughout all the session (traditional face-to-face modality).
- Practice with asynchronous distance feedback (ASYNC). The resident practiced alone, the instructor was not present and provided feedback asynchronously through the C1DO1 platform (Figure) (3).

Training consisted in 4 practice sessions, lasting one hour, distributed weekly. At the end of the training, both groups underwent a post-training evaluation (POST). PRE and POST assessments were videotaped to be evaluated by two independent and blinded reviewers, who rated the resident’s performance using a previous validated global rating scale (GRS) (4).

A non-parametric data distribution was assumed. Wilcoxon ranks and Wilcoxon signed ranks were used. Kappa coefficient was calculated for interobserver agreement. A p value of 0.05 was considered.

***Results & Discussion***

35 residents completed the training and both evaluations. Demographic data show no difference. Inter-rater reliability of the video GRS scores had a Cohen’s kappa of 0.81.

There were no differences between both groups in the PRE assessment, with a median of 28 points in the SYNC group and 26 in the ASYNC group (*p*= 0.56). Both groups significantly improved their GRS scores after four sessions: SYNC improved from 28 to 45 points (*p* < 0.01); the ASYNC group improved from 26 points to 46 points (*p* < 0.01). We found no significant differences between the groups in POST assessment (*p*= 0.31).

***Conclusion***

This simulation-based training program significantly improves residents PICC placement skills with both modalities (synchronous and asynchronous feedback). The asynchronous feedback training modality seems to be a comparable alternative to traditional face-to-face training methodologies, opening a new and innovative possibility for teaching procedural skills in healthcare.

***Keywords***

Procedural skills; PICC placement; vascular access; ultrasound-guided; simulation-based training; Distance-Based Simulation

***References***

1. Huang GC, McSparron JI, Balk EM, Richards JB, Smith CC, Whelan JS, Newman LR, Smetana GW. Procedural instruction in invasive bedside procedures: a systematic review and meta-analysis of effective teaching approaches. BMJ Qual Saf. 2016 Apr;25(4):281-94. 10.1136/bmjqs-2014-003518. Epub 2015 Nov 5. PMID: 26543067.
2. Corvetto M. Simulation-based training program with deliberated practice for ultrasound guided jugular central venous catheter placement. Acta Anaesthesiol Scand. 2017 Oct;61(9):1184-1191.
3. Vera M, Kattan E, Cerda T, Niklitshek J, Montaña R, Varas J, Corvetto MA. Implementation of Distance-Based Simulation Training Programs for Healthcare Professionals: Breaking Barriers During COVID-19 Pandemic. Simul Healthc. 2021 Dec 1;16(6):401-406. 10.1097/SIH.0000000000000550. PMID: 33913677.
4. Ma I, Zalunardo N, Pachev G, Beran T, Brown M, Hatala R. Comparing the use of global rating scale with checklists for the assessment of central venous catheterization skills using simulation. Adv Health Sci Educ Theory Pract 2012; 17: 457–70.

**Fig. 1 (Abstract O18).**
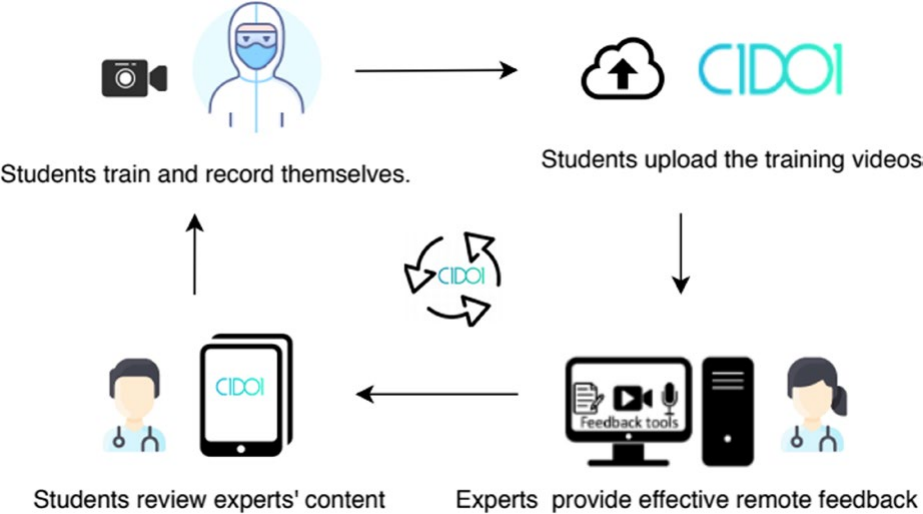
See text for description

## O19. Simulation based mastery learning for chest drain insertion: impact of booster session timing and interim clinical exposure on confidence for internal medicine trainees

Published article: https://bmcmededuc.biomedcentral.com/articles/10.1186/s12909-022-03654-7

*Adv Simul 2023*, **8(1)**:O19

## O20. Skills acquisition for newborn resuscitation after Helping Babies Breathe (HBB) simulation training in 12 health facilities across two regions in Tanzania – the SaferBirths Bundle of Care (SBBC) program

### Florence Salvatory Kalabamu^1^, Benjamin Kamala^2^, Robert Moshiro^1^, Hege Hersdal^3^, Rose Mpembeni^1^

#### ^1^Muhimbili University of Health and Allied Sciences; ^2^Haydom Lutheran Hospital; ^3^University of Stavanger

*Adv Simul 2023*, **8(1)**:O20

***Introduction***

Skilled newborn resuscitation of non-breathing babies is crucial for survival. Previous HBB skills training interventions using basic newborn simulator have shown immediate improvements in skills; however, skills decayed with time which is associated with poor newborn outcome. In order to address this gap, SBBC was introduced in five regions in Tanzania to reduce newborn deaths by conducting in facility low dose high frequency simulation-based skills training (LDHF-SBT) using improved training and clinical tools, and continuous quality improvement based on facility data. The aim of this study was to assess effects of simulation-based training using innovative tools on skills acquisition among HCWs immediately after a one-day training in Shinyanga and Geita regions, Tanzania.

***Methods***

A one-day facility-based training was conducted using the Helping Babies Breathe (HBB) 2nd edition HCWs in labor ward and obstetric theatres in 12 facilities across Geita and Shinyanga regions were trained using an improved newborn simulator (NeoNatalie Live), upright bag/mask resuscitator, and newborn heart rate monitor (NeoBeat). Skills scores were evaluated immediately after the one-day course using objectively structured clinical evaluation (OSCE) A and B which are WHO approved tools for assessing skills on routine newborn care and resuscitation developed by American Academy of Pediatrics. Data was analyzed using SPSS version 23.

***Results & Discussion***

A total of 479 HCWs were trained between November 2021 and January 2022. Immediately after the training, mean score for OSCE-A and OSCE-B were 92.2±8.3% and 92.8±6.1% respectively. Mean scores for OSCE A for health centers, district hospitals, and regional referral hospitals were 89.7±9.2%, 92.7±7.8%, and 93.7±7.6% respectively. There was a significant mean score difference across health facility levels (F (2) =8.9, *p*<0.001). On post hoc pairwise comparison, district and regional hospitals had significantly higher scores compared to health centers (*p*= 0.03 and *p*<0.001 respectively). Likewise, mean scores for OSCE-B were 91.7±6.7%, 93.7±4.6%, and 92.5±7.2% for health centers, district hospitals, and regional hospitals respectively. There was a significant difference in OSCE-B mean scores across health facility levels (F (2) =4.5, *P*=0.01. On post hoc pairwise comparison, district hospitals have significant higher mean score compared to health centers (*p*=0.01).

A simulation-based training using innovative tools was associated with adequate skills acquisition among HCWs. However, providers achieved different levels of skills between facilities with district and regional hospitals achieving higher scores than health centers. These findings indicate that SBT using improved tools is effective in newborn resuscitation skills acquisition among HCWs at baseline, and if LDHF-SBT is well implemented, has the potential to maintain the skills among HCWs. However, for the intervention to be more effective, further exploration of factors for differences across the facilities and mitigation strategies are warranted during the implementation of SBBC.

***Keywords***

Helping Babies Breathe, newborn, resuscitation, simulation

***Acknowledgements***

All supporting staff in the SaferBirths Bundle of Care, Geita, and Shinyanga Regional Medical Officers, and all healthcare providers who participated in the trainings

## O21. Students’ experiences with virtual reality simulation for soft skills in higher education for healthcare and social work

### Silje Stangeland Lie, Nikolina Helle, Miriam Dubland Vikman, Renate Westervik Alvestad, Tone Dahl Michelsen

#### VID specialized university

*Adv Simul 2023*, **8(1)**:O21

***Introduction***

Virtual reality (VR) provides students in higher education opportunities to practice complex and challenging situations without risking clients, patients or themselves. Thus, VR is gaining ground in clinical skills training in healthcare and medical education. However, a research gap exists regarding how VR simulation might facilitate non-technical and soft skills. This study is part of a larger interprofessional project that is developing, testing and evaluating a VR simulation programme for non-technical and soft skills for healthcare and social work students in a Norwegian higher education institution.

In the larger study, 360° movies were developed for students to watch on VR headsets for an immersive experience of different professional, demanding situations requiring advanced soft skills.

The full VR simulation consisted of a short brief, a 360° movie, and a debrief in groups of 5–8 students. The debrief was led by a facilitator and highlighted students’ reactions to the 360° movie, their considerations of the actions of the professional and the client/patient and their evaluations of their own future learning needs.

The aim of this study is to explore student’s experiences with VR simulation and how this learning design can stimulate their professional development.

***Methods***

The empirical data consisted of six focus group interviews with 28 bachelor’s students (nursing, social work, social education and occupational therapy) who participated in VR simulation. We used a qualitative design, and the transcribed interviews were analysed using thematic analysis.

***Results & Discussion***

According to the students, VR simulation was conducive to their professional development. They underscored that the immersive 360° movie, followed by team-based debriefing, facilitated emotions for learning. They experienced VR simulation as “alive and real” and a distinctive learning experience compared to case studies and textbooks.

Furthermore, it was experienced as a safe opportunity to explore different perspectives on complex and challenging situations. Notably, the students added the perspectives and observations of other students to their own. They considered this unique; according to how they differently from clinical placement all had experienced the same situation. Hence, VR simulation was experienced as a valuable didactic tool to prepare students to deal professionally with vulnerable patients or clients.

***Keywords***

Innovative pedagogy, virtual reality simulation, communication, soft skills, interprofessional, nursing students, social work students, social education students, occupational therapy students

**References**

1. Pottle, J., Virtual reality and the transformation of medical education. Future healthcare journal, 2019. 6(3): p. 181-185.
2. Hamilton, D., et al., Immersive virtual reality as a pedagogical tool in education: a systematic literature review of quantitative learning outcomes and experimental design. Journal of Computers in Education, 2021. 8(1): p. 1-32.
3. Allcoat, D. and A. von Mühlenen, Learning in virtual reality: Effects on performance, emotion and engagement. Research in Learning Technology, 2018. 26(0).
4. Woon, A.P.N., et al., Effectiveness of virtual reality training in improving knowledge among nursing students: A systematic review, meta-analysis and meta-regression. Nurse Education Today, 2021. 98.
5. Vesisenaho, M., et al., Virtual Reality in Education: Focus on the Role of Emotions and Physiological Reactivity. Journal For Virtual Worlds Research; Vol 12, No 1 (2019): Pedagogy - Taking Stock and Looking Forward (Part 2), 2019.
6. Tyng, C.M., et al., The influences of emotion on learning and memory. Frontiers in Psychology, 2017. 8: p. 1454-1454.
7. Beverly, E., et al., Perspectives of 360-Degree Cinematic Virtual Reality: Interview Study Among Health Care Professionals. JMIR Medical Education, 2022. 8(2): p. e32657.

**Fig. 1 (Abstract O21).**
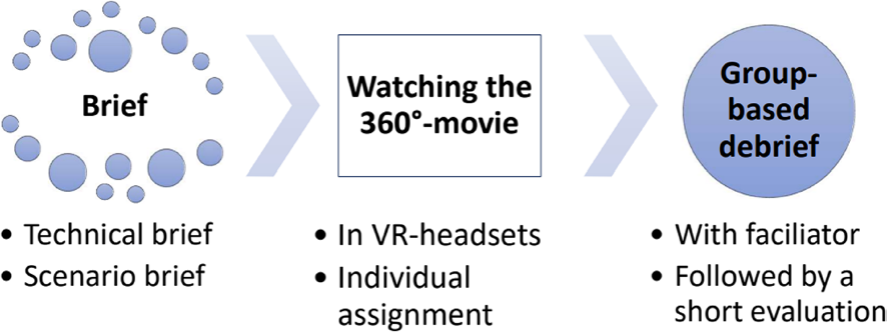
See text for description

## O22. The coroner’s court course

### Alina Cuhraja, Penelope Brown, Marta Ortega Vega, Megan Fisher

#### Maudsley Learning, South London and Maudsley NHS Foundation Trust

*Adv Simul 2023*, **8(1)**:O22

***Introduction***

One of the most challenging aspects of working in healthcare is experiencing a death of a patient who has been under your care. Often, the death is referred to the coroner to hold an inquest. This is an investigation into the cause of death and, in some circumstances considers what action can be taken to prevent future deaths. Being involved in a coroner’s inquest is a very real and anxiety-provoking prospect for many clinicians, yet there is no formal training provided for this.

***Methods***

This one-day interactive online course aimed to cover everything a clinician needs to know about the coroner’s court, including the impact on families, and relevant skills for the preparation and writing of reports and giving oral evidence. The course was piloted for psychiatry specialist registrars working in South London and Maudsley NHS Foundation trust. The course was led by a practicing Coroner, an experienced mental health lawyer, and a consultant psychiatrist, with invaluable contributions from families who have lost loved ones in mental health and custodial settings, and their legal representative.

The morning sessions focused on learning about the inquest process and the roles of different individuals involved. The afternoon sessions were interactive, including a report writing workshop and simulations of common coroner’s court scenarios. The example scenarios were a coroner taking a participant through a report, being questioned by a non-hostile Trust representative versus being questioned by a family representative. The modified Pendleton’s model was used for debriefing with feedback from all expert facilitators. Throughout the course, the emotional impact of inquests on clinicians and families and relevant support resources were considered.

***Results & Discussion***

The course was attended by 20 psychiatry registrars, aged from 30 to 55 years. Participants were asked to complete a pre-course and post-course questionnaire. The findings showed a significant increase in their understanding and confidence relating to different aspects and skills of the coroner’s inquest between the pre-course (M = 2.88, SD = 0.45) and post-course scores (M = 3.84, SD = 0.88), t(13) = 3.12, *p* = .008.The majority of participants identified the simulation component as the most helpful components of the course. Overall, the findings demonstrate the effectiveness of this course for supporting clinician’s understanding and confidence in undergoing a coroner’s court inquest. Simulation provides a valuable tool to prepare clinicians for this challenging aspect of their role through providing an environment for psychologically safe practice and development.

***Keywords***

mental health

**Acknowledgements**

Many thanks to Jacqueline Devonish (Coroner), Nigel Parsley (Coroner), Toby de Mellow (Trust’s solicitor), Raju Bhat (Families’ solicitor) and families for all their help in putting this course together.

## O23. The KUBS: a simulation tool box for training french psychiatric caregivers specialized in prehospital interventions during large scale sanitary events (CUMPS)

### Dominique Mastelli^1,2^, Pierre Vidailhet^1,2^

#### ^1^CUMP; ^2^Unisimes

*Adv Simul 2023*, **8(1)**:O23

***Introduction***

In 1995, the french government created the CUMPs, that is medical units specialized in pre-hospital psychiatric interventions during large scale sanitary events (i.e. terrorist attacks, natural or industrial disasters). CUMPS are distributed across the french territory with both permanent and voluntary healthcare professionals: secretaries, nurses, psychologists and psychiatrists. There is a major need for regular training to ensure a perfect fit when these caregivers have to intervene in stressful emergency situations, in natural areas, implicating collaboration with police, politics, medical emergency units. Each prefecture organizes full scale simulations once a year in real environments; however such simulations mobilize a large number of professionals and real structures (for instance a theater, a stadium, or an highway) and are time consuming.

***Methods***

We therefore created board games to train CUMP teams in reduced scale, but realistic, simulations of such exceptional sanitary events. We used a three drawers tool box, named the KUBS, to organize three levels of interventions: the first level uses an exact, but reduced-scale, reproduction of disaster places (for instance : a factory, a highway, a school) with small personages and vehicles testing interprofessional interactions scenario; the second level allows to mimic the organization of the psychiatric advance medical post for numerous victims; the third level gives learners scenario to provide individual full psychiatric care for involved victims.

***Results & Discussion***

Board games were judged as very efficient pedagogic tools by CUMP professionals, and the KUBS was evaluated both by trainers and learners as a useful and handy box, which adds a gaming feeling during training sessions.

***Keywords***

Psychiatry; emergency; CUMP; board games; KUBS

## O24. The constructivist template method: uses in simulation-based research

### Samantha Eve Smith, Victoria Ruth Tallentire

#### Scottish Centre for Simulation and Clinical Human Factors

*Adv Simul 2023*, **8(1)**:O24

***Introduction***

Constructivist methods are new to many healthcare professionals conducting simulation-based research (SBR). Thematic analyses are common constructivist data analysis methods. In SBR, thematic analyses are divided into four categories:(1) template analysis,(2) framework analysis,(3) thematic analysis(4) and qualitative content analysis.(5) Some of these methods recommend inductive analysis (in which codes are derived from the data), which is only appropriate when there is an absence of pre-existing literature. Others recommend deductive analysis (whereby pre-existing codes are applied), but fail to detail the underpinning philosophical assumptions. In order to produce high-quality research, novice researchers require clear, explicit methods with robust theoretical underpinnings. We argue in favour of an emerging method of thematic analysis that we believe is best suited to contemporary SBR: the constructivist template method.

***Methods***

Through conducting SBR over the last 12 years, including completion of our doctoral degrees, we have developed the constructivist template method. Our experience of conducting research using constructivist grounded theory (6,7), framework analysis(8) and template analysis(9) informed its development. Our reflections and discussions in the process of completing these studies resulted in the creation of the new method. The constructivist template method has informed the data collection and analysis of several recent projects.(10–13) It incorporates the problem/gap/hook heuristic,(14) use of conceptual frameworks,(15) purposive sampling and saturation of the themes from constructivist grounded theory,(16) deductive coding as in template analysis, and emphasises the generation of actionable outcomes as in framework analysis. It includes explicit methods of modifying the coding template to better fit the data. The stages of the constructivist template method are shown in the figure.

***Results & Discussion***

Arguments in favour of using the constructivist template method for SBR include the fact that it helps build on existing theory, rather than looking at the data in isolation. It incorporates the foundations of constructivism, as described by Charmaz.(16) It is well suited for SBR, because it helps to move the researcher from abstract conceptualism to practical uses of the theories that are built. Limitations of this method are similar to other constructivist research methods, in that the results are not usually generalisable, but may be transferable to other contexts.(16) The constructivist template method may appeal to simulation-based researchers who are new to constructivism, as it is contains a clear step-wise approach, justification for data collection and analysis methods, and helps guide researchers towards actionable outcomes.

***Keywords***

Research, Constructivism, Thematic Analysis, Methods

***References***

1. Nestel D, Hui J, Kunkler K, Scerbo MW, Calhoun AW. Healthcare simulation research. Springer; 2019.
2. King N. Template Analysis. In: Symon G, Cassell C, editors. Qualitative Methods and Analysis in Organizational Research: A Practical Guide. London: SAGE Publication; 1998. p. 118–34.
3. Ritchie J, Spencer L. Qualitative data analysis for applied policy research. In: Bryman A, Burgess R, editors. Analyzing Qualitative Data . London and New York: Routledge; 1994. p. 173–94.
4. Braun V, Clarke V. Using thematic analysis in psychology. Qual Res Psychol. 2006;3(2):77–101.
5. Hsieh HF, Shannon SE. Three approaches to qualitative content analysis. Qual Health Res. 2005;15(9):1277–88.
6. Tallentire VR, Smith SE, Skinner J, Cameron HS. Understanding the behaviour of newly qualified doctors in acute care contexts. Med Educ. 2011;45(10):995–1005.
7. Smith SE, Tallentire VR, Cameron HS, Wood SM. The effects of contributing to patient care on medical students’ workplace learning. Med Educ. 2013;47(12):1184–96.
8. Tallentire VR, Smith SE, Skinner J, Cameron HS. Exploring patterns of error in acute care using framework analysis. BMC Med Educ. 2015;15(1):1–8.
9. Tallentire VR, Smith SE, Skinner J, Cameron HS. Exploring error in team-based acute care scenarios: an observational study from the United Kingdom. Academic Medicine. 2012;87(6):792–8.
10. Kerins J, Smith SE, Stirling SA, Wakeling J, Tallentire VR. Transfer of training from an internal medicine boot camp to the workplace: enhancing and hindering factors. BMC Med Educ. 2021;21(1):1–12.
11. Smith SE, Tallentire VR, Pope LM, Laidlaw AH, Morrison J. Foundation Year 2 doctors’ reasons for leaving UK medicine: an in-depth analysis of decision-making using semistructured interviews. BMJ Open. 2018;8(3):e019456.
12. Kerins J, Smith SE, Phillips EC, Clarke B, Hamilton AL, Tallentire VR. Exploring transformative learning when developing medical students’ non technical skills. Med Educ. 2020;54(3):264–74.
13. Tallentire VR, Smith SE, Facey AD, Rotstein L. Exploring newly qualified doctors’ workplace stressors: an interview study from Australia. BMJ Open. 2017;7(8):e015890.
14. Lingard L, Watling C. Problem/Gap/Hook Introductions. In: Story, Not Study: 30 Brief Lessons to Inspire Health Researchers as Writers. Springer; 2021. p. 7–14.
15. Bordage G. Conceptual frameworks to illuminate and magnify. Med Educ. 2009;43(4):312–9.
16. Charmaz K. Constructing grounded theory: A practical guide through qualitative analysis. sage; 2006.

**Fig. 1 (Abstract M1).**
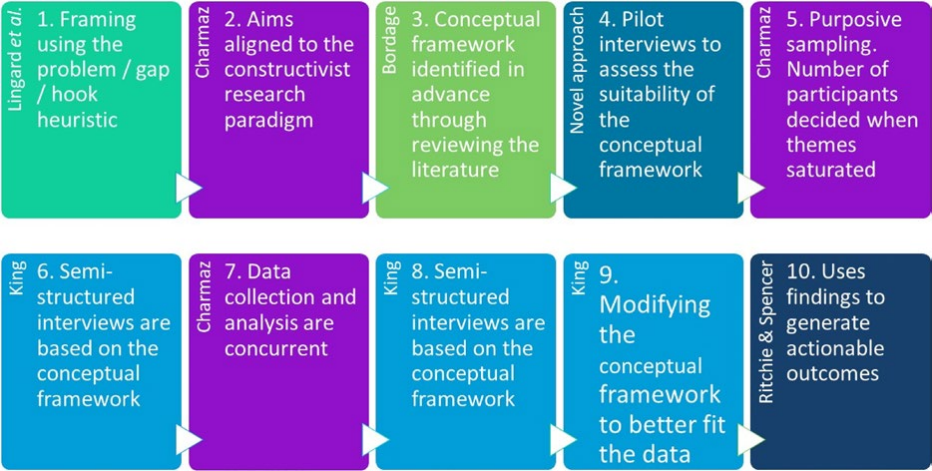
See text for description

## O25. The effect of deception in simulation-based education in healthcare: a systematic review and a meta-analysis

 Published article: https://www.ijohs.com/article/doi/10.54531/hwxl4351#

*Adv Simul 2023*, **8(1)**:O25

## O26. Training foundation doctors in management of sexual assault: a pilot simulation

### Ellen Rhodes, Peter Kilgour

#### Salford Royal Hospital, Northern Care Alliance

*Adv Simul 2023*, **8(1)**:O26

***Introduction***

In the UK, 1 in 4 women and 1 in 20 men have been raped or sexually assaulted in adulthood1. Healthcare professionals are likely to encounter this presentation particularly in the Emergency Department (ED), yet it is rarely included in medical school curriculum or UK foundation programme training2. Simulation education involving this topic has been used to effectively train nurses and ED doctors in America3-5 but has not been adequately explored in UK foundation trainees.

***Methods***

A pilot session was designed to simulate the management of an adult female attending ED after sexual assault.

Pre-session learning resources were distributed to participants and resources for personal support were provided at every stage. The session consisted of three groups of six doctors in their second-year post-qualification. One doctor engaged directly with an actor while the group observed separately via an audio-visual feed. A post-session debrief with the actor, participants and a subject matter expert discussed the importance of non-judgemental history taking, non-verbal communication skills and the use of empathy in this scenario. The group also explored the reasoning behind any management advice provided to the victim during the scenario and feedback on performance gaps was provided.

15 participants completed pre- and post-simulation feedback forms, which asked doctors to rank confidence in four aspects on a scale of one to ten and provided opportunity to leave free text comments on the session.

***Results & Discussion***

This topic is not compulsory within the UK foundation programme or medical school curricula. This is reflected in our results as only two out of the 15 surveyed had prior relevant teaching. Post-session, participants felt significantly more confident in eliciting a history, patient management and understanding the role of SARC (all p<0.001, paired t test). In free text comments, participants emphasised the importance of the debrief, remarked upon excellent pre-session material and praised the use of a patient actor. They also commented that the session improved both medical knowledge and communication skills. Recurring themes in suggestions for improvement were more time to complete the session and more opportunities for wider participation in the scenario itself.

The authors feel that this topic is highly relevant but sadly neglected in current training. This study provides evidence that the use of simulation education can be utilised to improve the knowledge, skills and behaviours required to effectively manage a clinical presentation of sexual assault in an ED.

***Keywords***

Emergency Department

**Acknowledgements**

Thank you to Carole Gavin (Emergency medicine consultant at Salford Royal Hospital) for providing advice on relevant resources.

**References**

1. Office for National Statistics (ONS), released 18 March 2021, ONS website, dataset, Title: Sexual offences prevalence and victim characteristics, England and Wales
2. UK Foundation Programme Office. UK Foundation Programme Curriculum 2021 [Internet]. London: UKFPO; 2021 [cited 2022 Oct 14]. Available from: https://healtheducationengland.sharepoint.com/:b:/s/UKFPOT/EQcs_1YtgdhNlDMRJoBGP74Bk9i1ICDnzHrj8RD3VpuW-w?e=JwX7ZI.
3. Bechtel K, Bhatnagar A, Joseph M, Auerbach M. Sexual Assault in an Adolescent Female: A Pediatric Simulation Case for Emergency Medicine Providers. MedEdPORTAL. 2020;16:10942.
4. Nathan S, Moret JD. Sexual Assault Forensic Examiner Recruitment and Retention: Using Simulation to Teach a Trauma-Informed Interview. J Forensic Nurs. 2022;18(1):54-8.
5. Scannell M, Lewis-O'Connor A, Barash A. Sexual Assault Simulation Course for Healthcare Providers: Enhancing Sexual Assault Education Using Simulation. J Forensic Nurs. 2015;11(4):188-97.

## O27. Transforming professional identity in simulation debriefing: a systematic meta-ethnographic synthesis of the simulation literature

### Ranjev Kainth, Gabriel Reedy

#### King’s College London

*Adv Simul 2023*, **8(1)**:O27

***Introduction***

Debriefing is extensively incorporated in simulation-based education, and there is some evidence relating debriefing to participant learning. However, there is a lack of detailed understanding of how debriefing works and how it enables learning, specifically examining participant-faculty interactions. The existing research which has examined interaction in simulation debriefs is heterogenous in nature and published across different research disciplines, making holistic interpretations of the phenomenon challenging. To further our understanding and simultaneously illuminate current knowledge, a qualitative synthesis is required.

***Methods***

A meta-ethnography qualitative synthesis was undertaken using Noblit and Hare’s seven phase approach to address the research question: how are interactions in simulation debriefing related to participant learning? Searching strategy was based upon the PICOS framework; 10 databases were searched which concluded in November 2020. Included articles were limited to facilitator-guided post-event debriefing. Selected studies were read multiple times and the interpretations of authors of those studies were synthesised to produce new concepts which were further interpretated to produce a new overarching framework to explain the process of learning in simulation debriefs. Data was also extracted in relation to contextual and demographic factors, and to enable quality appraisal which facilitated interpretation and contextualisation of the final findings.

***Results & Discussion***

Results

Seventeen articles were selected for inclusion. Initial interpretive synthesis generated 37 new translations which were further synthesised to produce a new theoretical framework. At the heart of the framework is a concept of reflective work where participants and faculty recontextualise the simulation experience, bidirectionally with clinical reality: a process which facilitates sense-making. This occurs in a learning milieu where activities such as storytelling; performance evaluation; perspective sharing; agenda setting; and video use are undertaken. The learning milieu can also be influenced by purposeful activities which relive and reconstruct the simulation experience. The outcome of recontextualisation in a psychologically safe space is conceptualisation of new future roles, clinical competence, and professional language development; a process of transforming professional identity.

Discussion

The framework materialises the interactions which take place in debriefings and describe how these are related to the learning process. Aspects of the framework are related to existing literature including research from different methodological positions, and learning theories, particularly Kolb and Mezirow. This work opens further domains for research including in settings not yet undertaken and thus not represented in the framework, and secondly, research linked directly to the framework.

***Keywords***

debrief; learning; qualitative review; synthesis; meta-ethnography; simulation; interaction

**Acknowledgements**

Dr Anne McKee provided supervisory support to the first author.

**Fig. 1 (Abstract O27).**
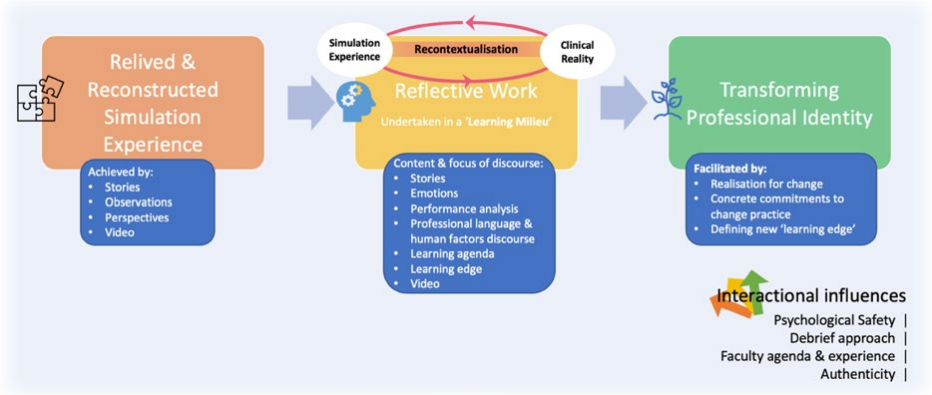
See text for description

## O28. Understanding trauma and its impacts on children and adolescents’ mental health: a blended learning course for mental health professionals

### Naila Saleem, Marcela Schilderman, Valerie Hartland, Marta Ortega Vega, Kiran Virk

#### Maudsley Learning, South London and Maudsley NHS Foundation Trust

*Adv Simul 2023*, **8(1)**:O28

***Introduction***

A significant number of children and adolescents are exposed to traumatic life events and are likely to develop PTSD. In community samples, more than two thirds of children report experiencing a traumatic event by age 16 (1). It is imperative that people working with children and adolescents feel confident and have the relevant knowledge and skills in using trauma informed practice.

***Methods***

This was a 2-day blended learning course designed for mental health professionals working in community Child and Adolescent Mental health Services in Doncaster. The course was delivered face to face and composed of pedagogy and immersive simulation scenarios. The aim of the course was to develop their confidence, knowledge and skills in trauma informed practice. The day involved half a day of didactic teaching followed by three simulated scenarios to enable participants to practice skills learned from the didactic teaching. 10 participants attended the course including a psychologist, CAMHS practitioners, CAMHS nurses, a drama therapist, and a pathway lead.

Psychological safety was first established by group icebreakers and didactic introduction to simulation training, followed by 6 scenarios (3 scenarios each day) including suicidality, self-harm in the context of sexual abuse and difficult family dynamics, eating disorder, complex PTSD, aggression and suspected drug use. The

Maudsley debrief model was used to provide feedback to participants on their contributions and help them learn positively and constructively from the experience, enabling a deeper level of learning, reflection, and group discussions. It covered description, analysis, and application over the course of 40 minutes.

***Results & Discussion***

Participants were asked to complete a post-course questionnaire to evaluate their learning and satisfaction with the course. Of 7 participants who completed this, 86% of participants responded “Good” or “Excellent” to how well the course met its learning objectives, the relevance of the scenarios, and usefulness of the course for their practice. Additionally, qualitative data highlighted learning in relation to the importance of debriefs and reflective practice, as well as assessing and intervening in trauma in young people. Finally, participants shared various changes they intend to make to their practice post-course, including creating more spaces to reflect with colleagues, recognising the non-verbal indicators of trauma, and using creative strategies to engage the child.

The results demonstrate that the course was effective in enhancing both knowledge and skills, as well as helping the participants in becoming confident in using trauma informed practice.

***Keywords***

simulation; mental health; children and young people; PTSD

***Reference***

1. American Psychological Association (2008). Children and Trauma Update for Mental Health Professionals. [Retrieved from: https://www.apa.org/pi/families/resources/children-trauma-update#:~:text=In%20community%20samples%2C%20more%20than%20tw

## O29. Unintended consequences of simulation: a framework analysis of the translational outcomes from a simulation programme for early-stage doctors-in-training

### David MacLennan, Vicky Tallentire, Nathan Oliver

#### NHS Lothian

*Adv Simul 2023*, **8(1)**:O29

***Introduction***

Simulation aims to produce ‘positive consequences’ but the literature warns that simulation may promote over-confidence that goes beyond the practitioners expected level of proficiency: the Dunning-Kruger effect (1,2). This has been reported in medical students where learners undergoing high-fidelity simulation rated their confidence levels greater than peers who did not, with no difference in objective assessment between groups (3). This effect has been further evidenced in a study comparing learners’ outcomes in simulation-based education (SBE) to case-based discussion (4). Despite this, there is limited research on how this may translate to early-stage Doctors-in-Training (DIT) operating beyond their competency, a significant patient safety concern.

Aspects of communication and escalation of care are key ‘non-technical’ learning outcomes of many existing healthcare simulation programmes. Yet, both nationally and internationally, these are consistently identified as the main causes of complaints and negative patient outcomes (5-7).

This study aimed to explore the unintended consequences of a simulation programme for DIT. Specifically, do early-stage DIT use skills learned in simulation beyond their expected competency in relation to communication and escalation of care?

***Methods***

All early-stage DIT (Foundation year one [FY1]) were invited to participate in an immersive simulation programme across three hospital sites in NHS Lothian, Scotland. Following ethical approval, a voluntary questionnaire was sent to participants after their immersive simulation session, asking them to describe two clinical cases where the programme had directly influenced their clinical practice. Over a five-year period, 528 case reports from 264 individuals were collated and analysed using a deductive framework generated from a combination of the scenario intended learning outcomes and the FY1 curriculum, focusing on communication and escalation of care.

***Results & Discussion***

Escalation of care was consistently utilised appropriately in clinical practice (“I called for help early”) in DIT questionnaires. In contrast, respondents reported adopting the communication skills taught in simulation but reported applying them in inappropriate contexts and without appropriate supervision. Some actions described in clinical practice went beyond the level of expected competency (“to the patient and family, that his cancer was progressing”). Consistent escalation of care is a positive outcome as delays in escalation are associated with an increased incidence of negative outcomes (7,8). However, the communication findings have serious implications for SBE; specifically, how early-stage DIT are taught to escalate challenging communication scenarios and how they acknowledge their own limitations to mitigate unintended consequences. Further work will focus on facilitators and barriers to DIT good communication practices.

***Keywords***

Medicine, Simulation, Communication, Escalation of care

***References***

1. Kruger J, Dunning D Unskilled and unaware of it: How difficulties in recognising one’s own incompetence lead to inflated self-assessments Journal of personality and social psychology 1999 77 (6): 1121-1134
2. Winner J Millwater T Evaluating human patient simulation fidelity and effectiveness for combat-medical training Proceedings of the International Symposium on Human Factors and Ergonomics in Health care 2019; 8 (1):176-180
3. Massoth, C., Röder, H., Ohlenburg, H. et al. High-fidelity is not superior to low-fidelity simulation but leads to overconfidence in medical students. BMC Medical Education 2019 19, 29 (2019). 10.1186/s12909-019-1464-7.
4. Wenk M, Waurick R, Schotes D, Wenk M, Gerdes C, Van Aken HK, Popping DM Simulation-based medical education is no better than problem-based discussions and induces misjudgement in self-assessment Advances in health sciences education: Theory and practice 2009 14 (2):159-171
5. NMAHP-RU A scoping review of evidence relating to communication failures that lead to patient harm [internet] 2018 [cited 2022] available from: https://www.gmc-uk.org/-/media/documents/a-scoping-review-of-evidence-relating-to-communication-failures-that-lead-to-patient-harm_
6. Raberus A, Holstrom I, Galvin K, Sundler A The nature of patient complaints: a resource for healthcare improvements International Journal for quality in Health Care 2018; 31 (7): 556-562
7. O’Neill SM, Clyne B, Casey A, Leen B, Smith SM, Ryan M, O’Neil M Why do healthcare professionals fail to escalate as per the early warning system (EWS) protocol? A qualitative evidence synthesis of barrier and facilitators of escalation BMC Emergency medicine 2021; 21 (15): 1-19
8. Smith GB In-hosptial cardiac arrest: Is it time for an in-hospital chain of prevention Resuscitation 2010 81: 1209-1211

## O30. Using immersive simulation for antimicrobial stewardship

### Daniel Slack, Caity Irvine, Stephanie Dundas

#### NHS Lanarkshire

*Adv Simul 2023*, **8(1)**:O30

***Introduction***

Antimicrobial Stewardship programmes (ASPs) are widely used to improve antimicrobial resistance awareness and educate healthcare professionals about good principles of antimicrobial usage. The Scottish Antimicrobial Prescribing Group (SAPG) developed the Hospital Antibiotic Review Programme (HARP), which sets out key principles of antimicrobial prescribing to reduce the burden of resistance. Although ASPs deliver increased knowledge, evidence from local stewardship teams shows this does not always translate into practice.

High-Fidelity simulation (HFS) is ubiquitous in medical education and a key advantage is generation of behaviour change.

We aimed to design a novel ASP using HFS which would generate increased behaviour change with respect to antimicrobial prescribing.

***Methods***

We designed a HFS Ward Round, with three low-acuity simulated patients that specifically targeted antimicrobial stewardship principles, including those highlighted by HARP. Simulated Patients were played by actors, and realistic documentation was provided for each patient. Prescribing was done electronically.

Following a pre-brief, participants completed a ward round of the simulated patients in a 40 minute scenario. They were then debriefed about the decisions they made, how they made them and how this will impact the patient.

***Results & Discussion***

To the authors’ knowledge, no similar programme using HFS to address antimicrobial stewardship issues exists currently. We analysed the effect of this programme on self-reported confidence, knowledge acquisition and actual prescribing behaviour.

In total, 14 first year foundation doctors took part. Pre- and post-course MCQ tests showed a mean improvement of 84.5% overall, which was statistically significant (*p* = <0.001).

Pre- and post-course questionnaires assessing self-reported confidence showed that 100% of participants improved in their overall confidence regarding the prescription of antimicrobials, including interpreting culture results and guidelines. Additionally, 100% of candidates agreed that simulation was both a valid and enjoyable way to learn about antimicrobial stewardship.

Analysis of online prescribing data is ongoing, but initial analysis suggests that overall prescribing has improved after the course, particularly in the areas of IV to Oral Switch therapy and use of culture results and guidelines to aid prescribing. HFS is an effective way to teach antimicrobial stewardship principles and increases confidence and knowledge in different areas of antimicrobial prescribing. It also generates real change in terms of antimicrobial prescribing, which is of benefit to patients.

***Keywords***

Antimicrobial Stewardship

***Reference***

1. Hospital Antibiotic Review Programme (HARP) - accessed 14/09/22 at https://www.sapg.scot/guidance-qi-tools/quality-improvement-tools/hospital-antibiotic-review-programme-harp/

## O31. Ventilation effiectivness during paediatric cardiopulmonary resuscitation: simulation-based comparative study

### Tamara Skrisovska, Petr Stourac, Tereza Kramplova, Martina Kosinova

#### Department of Simulation Medicine, Faculty of Medicine, Masaryk University

*Adv Simul 2023*, **8(1)**:O31

***Introduction***

This prospective simulation-based comparative study aimed to evaluate the efficacy of ventilation during simulated paediatric cardiopulmonary resuscitation (CPR) provided by health care professionals (HCP) and lay rescuers (LR). European Resuscitation Council (ERC) guidelines 2021 were considered standard of care. The primary outcome was the number of effective breaths out of 5 initial CPR breaths before and after structured training. The secondary outcomes were: a sub-analysis of 2 first ventilation attempts, the time to first effective ventilation, and the time to the beginning of chest compressions. We hypothesised, that in HCPs more than 80% of ventilation attempts from 5 initial breaths will be effective (4 out of 5 breaths). In LRs, more than 60% of ventilation attempts (3 out of 5 breaths) and there will be a significant improvement in the number of effective ventilation in both groups after simulation-based training.

***Methods***

HCP and LR performed 90 seconds of CPR on 2 simulation mannequins: 5 kg Baby and 20 kg Junior. The HCPs provided bag-mask ventilation; LR performed dispatcher-assisted CPR with mouth-to-mouth ventilation. The effectiveness of ventilation (defined as a visible chest rise) was recognized by mannequin software and visually confirmed by trained independent observers (both had to be in concordance to mark a ventilation attempt as effective). The mannequin software obtained measurements of each breath time and chest compressions initiation.

***Results & Discussion***

Data were obtained from 40 HCPs and 46 LRs. Significant improvement was detected in the number of effective ventilations in Baby in HCP before and after the training 26 (65%) vs. 40 (100%), respectively and in LRs 28 (60.9%) vs. 45(97.8%), (both P 0.001). The improvement before and after the training in Junior was significant only in the LR group [32 (82.1%) vs. 37 (94.9%), *P*=0.005], not in HCP group [31 (77.5%) vs. 32 HCP (82.1%), (*P*=0.77)]. ERC guidelines recommendation to start ventilation with 5 initial breaths in paediatric CPR, was based on the experts´ opinion. (1) Other resuscitation guidelines worldwide do not include initial resuscitation breaths in lay rescuers. (2) (3) To our knowledge, this is the first study investigating ventilation effectiveness in paediatric CPR. Our data confirmed that lay rescuers providing dispatcher-assisted CPR are able to deliver effective breaths for both Baby and Junior. Obtained data also displays the benefit of practical paediatric CPR training by improving the number of effective initial breaths in both HCPs and LRs.

***Keywords***

paediatric, emergency medicine, CPR

Supported by MUNI/A/1105/2022 and MUNI/A/1109/2022/

***References***

1. Van de Voorde P, Turner NM, Djakow J, de Lucas N, Martinez-Mejias A, Biarent D, et al. European Resuscitation Council Guidelines 2021: Paediatric Life Support. Resuscitation. April 2021;161:327–87.
2. Maconochie IK, Aickin R, Hazinski MF, Atkins DL, Bingham R, Couto TB, et al. Pediatric Life Support: 2020 International Consensus on Cardiopulmonary Resuscitation and Emergency Cardiovascular Care Science With Treatment Recommendations. Circulation [Internet]. 20. 10. 2020 [cited 10.10. 2022];142(16_suppl_1). Available from: https://www.ahajournals.org/doi/10.1161/CIR.0000000000000894
3. The ARC Guidelines - Australian Resuscitation Council [Internet]. [cited 10. 10 2022]. Available from: https://resus.org.au/the-arc-guidelines/.

## O32. Which specific team behaviours influence successful outcome in internal medicine simulated cardiac arrest resuscitation?

Published article: https://www.ijohs.com/article/doi/10.54531/cope7296#

*Adv Simul 2023*, **8(1)**:O32

